# Pressure and Cross-Flow Dependent Interfacial Fouling of Mesoporous PES-PUMA Membranes During Water-in-Oil Emulsion Separation

**DOI:** 10.3390/membranes16100350

**Published:** 2026-10-21

**Authors:** Alireza Bahramian, Sedigheh Alavinia

**Affiliations:** 1 Chemical Engineering Department, Hamedan University of Technology, Hamedan 65155, Iran; 2 Department of Organic Chemistry, Faculty of Chemistry and Petroleum Sciences, Bu-Ali Sina University, Hamedan 65155, Iran

**Keywords:** modified polyethersulfone membrane, mesoporous hydrophobic hybrid structure, operating pressure and cross-flow effects, water-in-oil emulsion, interfacial fouling

## Abstract

Water-in-oil (W/O) emulsion treatment is challenging because efficient membrane separation requires simultaneous control of oil transport, water rejection, interfacial interactions, and fouling resistance. However, the coupled effects of operating pressure and cross-flow hydrodynamics on membrane pore accessibility, foulant-layer evolution, and the permeability-rejection trade-off during cyclic W/O emulsion treatment remain insufficiently understood. In this study, an electrospun mesoporous polyurethane–melamine/polyethersulfone (PES-PUMA) membrane was evaluated for the separation of stabilized W/O emulsions under different operating pressures and feed flow rates. At an ultra-low pressure of 20 kPa, the membrane achieved an oil permeate flux of 36.5 ± 2.7 L m^−2^ h^−1^ and water rejection of 93.1 ± 2.1%. Increasing the operating pressure to 100 kPa increased the oil permeate flux to 87.6 ± 3.5 L m^−2^ h^−1^ while decreasing water rejection to 78.2 ± 1.8%. Increasing the cross-flow rate from 0.05 to 0.5 mL s^−1^ increased the oil permeate flux from 23.4 ± 0.7 to 90.7 ± 2.1 L m^−2^ h^−1^ while decreasing water rejection from 95.3 ± 2.1% to 78.4 ± 1.2%. Quadratic regression models revealed significant nonlinear relationships between hydraulic operating conditions and both separation responses, providing a quantitative description of the pressure- and flow-dependent performance trade-off. Higher cross-flow reduced foulant-layer thickness and favored more reversible fouling, whereas increasing operating pressure promoted pore constriction and compact foulant deposition. These findings establish a quantitative pressure–cross-flow–transport–fouling relationship, demonstrating that hydraulic conditions jointly regulate membrane permeability, water rejection, foulant-layer development, and fouling reversibility. This study therefore provides mechanistic and quantitative insight into optimizing the operation of the established PES–PUMA membrane to balance separation performance and fouling resistance during W/O emulsion treatment.

## 1. Introduction

Membrane-based separation has emerged as an attractive engineering strategy for oily wastewater treatment, particularly in applications that require the simultaneous achievement of efficient phase separation, oil recovery, and sustainable process operation [1,2,3]. Conventional separation methods, including oil skimming, adsorption–filtration, gravity settling, coagulation–flotation, and centrifugation, enable preliminary separation but are generally constrained by low efficiency, high energy consumption, secondary pollution, and limited oil recovery capacity.

These conventional methods are generally ineffective for treating stable emulsions with small droplet sizes and complex interfacial interactions. Subsequently, hydrophobic membranes have been developed using various engineering strategies, including structural optimization [4], the formation of nanoscale hierarchical structures [5], the incorporation of multifunctional polymers [6,7], and advanced surface modification techniques [8,9]. These advancements aim to enhance the interfacial affinity, surface wettability, and antifouling properties of the membranes [10]. Accordingly, the development of green, energy-efficient, and scalable separation technologies for complex W/O emulsions and resource recovery is an urgent priority.

The high kinetic stability of surfactant-stabilized emulsions, primarily caused by high interfacial tension and droplet coalescence resistance, adversely affects the separation of W/O emulsions [11]. Consequently, synthesizing membranes capable of stable W/O emulsion separation remains a significant challenge.

The effect of operating pressure on the separation of W/O emulsions has been highlighted in the literature [12]. The high viscosity of the oil phase can adversely affect mass transport and permeate flux in the absence of operating pressure [12]. Hydraulic pressure plays a critical role in accelerating permeation and overcoming the critical capillary entry pressure (Laplace pressure) of dispersed phase. However, excessive pressure can cause two major problems: droplet deformation and pore blockage, which result in reduced oil permeability and water rejection, respectively. Despite this, the relationship among membrane structure, pressure, and separation performance in W/O separation remains poorly understood in the literature [13,14,15]. In addition, the dispersed phase content significantly influences the stability of W/O emulsions. A higher water content increases droplet–droplet and droplet–membrane interactions, which promote droplet coalescence and pore blockage at higher water contents, ultimately reducing separation stability. Therefore, evaluating membrane performance across a broad range of water contents is essential for understanding its practical applicability under realistic W/O conditions [16].

Polymers are widely used in membrane formulations because of their film-forming ability, multi-functional groups, mechanical stability, and adjustable surface properties [17]. PES has attracted considerable interest because of its hydrophobic nature, excellent chemical stability, and mechanical strength. However, the currently available PES-based membranes reported to date are limited by their inadequate pore structures, fouling, and insufficient mechanical robustness. The lack of hierarchical porous frameworks combining macropores and micropores increases oil transport resistance and restricts separation flux. Nevertheless, most PES membranes have been designed primarily for O/W emulsion separation, with relatively limited application to W/O emulsions. Therefore, the engineering of PES-based membranes with tailored hydrophobic and oleophilic structures for effective W/O separation remains a key research challenge.

Electrospun nanofibrous membranes are ideal for designing membranes with a high surface area, tunability, and interconnected channels [18]. To enhance surface properties such as wettability and functionality, blending functional nanofillers into the polymer matrix represents an effective and straightforward strategy [19,20]. However, weak interfacial interactions between the fillers and polymer matrix can lead to a reduced chemical stability and long-term durability [10,21,22].

Recent research has focused on developing robust and recyclable membranes capable of efficiently separating W/O emulsions under realistic operating conditions to address these limitations. Many high-performance membranes are based on poly(vinylidene fluoride) (PVDF) that exhibit excellent oil affinity. However, their application poses risks to marine ecosystems and freshwater resources because of the production of hazardous byproducts. Moreover, complex fabrication processes limit their scalability for treating oily wastewater in industrial settings [23]. Therefore, the development of fluorine-free membranes for W/O emulsion separation has become a critical research priority.

Porous organic polymers (POPs) have been extensively used for membrane modification. In particular, porous polyurethane-based materials have not been investigated as functional modifiers for PES-based membranes in W/O emulsion separation. Polyurethane–melamine (PUMA)’s structure is particularly attractive due to its strong hydrogen-bonding interactions, structural flexibility, and enhanced thermal stability [12]. This combination could potentially improve membrane robustness, oil transport pathways, and resistance to water intrusion.

Despite recent advances in membrane materials for emulsion separation, fluorine-free and scalable PES membranes specifically designed for stable W/O emulsion separation under realistic water content, hydraulic pressure, and thermal recovery conditions remain scarce. Notably, the incorporation of PUMA into electrospun PES membranes has not yet been explored. The incorporation of PUMA into the PES matrix introduces structural heterogeneity and modifies the recyclability and morphological properties of the membrane while enhancing the hydrophobic porous structure and compatibility necessary for W/O emulsion separation [12]. The previously established structural characteristics, interfacial compatibility, and hydrophobicity of the PES–PUMA membrane support its selection as a platform for investigating pressure- and feed flow rate-dependent separation and fouling behavior. Therefore, this study focuses on the process-level effects of hydraulic operating conditions rather than on comparative screening of different composite modifiers.

Additionally, membrane thickness is a critical factor influencing the mass transport resistance, mechanical durability, and pressure tolerance of fibrous membranes. Our previous work established the fabrication and initial characterization of PES–PUMA membranes for W/O emulsion separation [12]. However, the effects of hydraulic operating conditions and repeated filtration on their separation performance, fouling evolution, and structural stability remain insufficiently understood.

To address these challenges, the present study evaluates the effects of operating pressure, cross-flow rate, and cyclic operation on the performance, fouling, and structural relationships of the previously developed PES–PUMA membrane. PUMA was synthesized and incorporated into the PES matrix, and the resulting PES–PUMA nanocomposite membranes were fabricated by electrospinning. The specific objectives of this study are to (1) synthesize and fabricate PES–PUMA membranes with tailored surface roughness, interconnected porosity, and suitable hydrophobicity for selective oil transport; (2) systematically characterize their morphology, surface roughness, pore structure, porosity, and thermal stability; (3) evaluate W/O emulsion separation performance over a dispersed-water content of 10 vol%; (4) establish structure–property–performance relationships to elucidate how PUMA incorporation regulates membrane morphology, oil transport, and water intrusion; and (5) systematically investigate the effects of transmembrane pressure (20–100 kPa) and feed flow rate (0.05–0.50 mL s^−1^) on oil permeate flux, water rejection, fouling evolution, and flux recovery, thereby identifying the hydraulic operating conditions that balance separation efficiency, permeability, and long-term fouling resistance. Unlike our previous work, which primarily focused on membrane development and initial characterization, the present study examines how hydraulic operating conditions influence the permeability–rejection trade-off, fouling evolution, and post-use structural changes. This approach provides a process-level understanding of the relationships between membrane structure, operating conditions, and fouling behavior during W/O emulsion separation.

## 2. Materials and Methods

### 2.1. Materials

The synthesis of SiO_2_ NPs is based on hydrolysis and condensation reactions of tetraethyl orthosilicate (TEOS) in the presence of ammonia solution and ethanol. Porous PUMA was synthesized according to our previous work [12]. The stabilized W/O emulsion was prepared following our previously reported method [12]. A summary list of chemicals and materials, along with their purities, employed in membrane fabrication and emulsion preparation is presented in Supplementary Materials (Table S1).

### 2.2. Fabrication of PES–PUMA Membrane

Mesoporous PUMA was synthesized using spherical SiO_2_ nanoparticles (20–80 nm) as a sacrificial template, prepared according to the Stöber method [24]. The silica template was subsequently removed by selective HF-assisted etching, followed by repeated washing and drying to obtain the mesoporous PUMA framework. The detailed synthesis, template-removal procedure, and morphological characterization of the SiO_2_ nanoparticles are provided in the Supplementary Material (Section S2.1 and Figure S1).

**Scheme 1:**
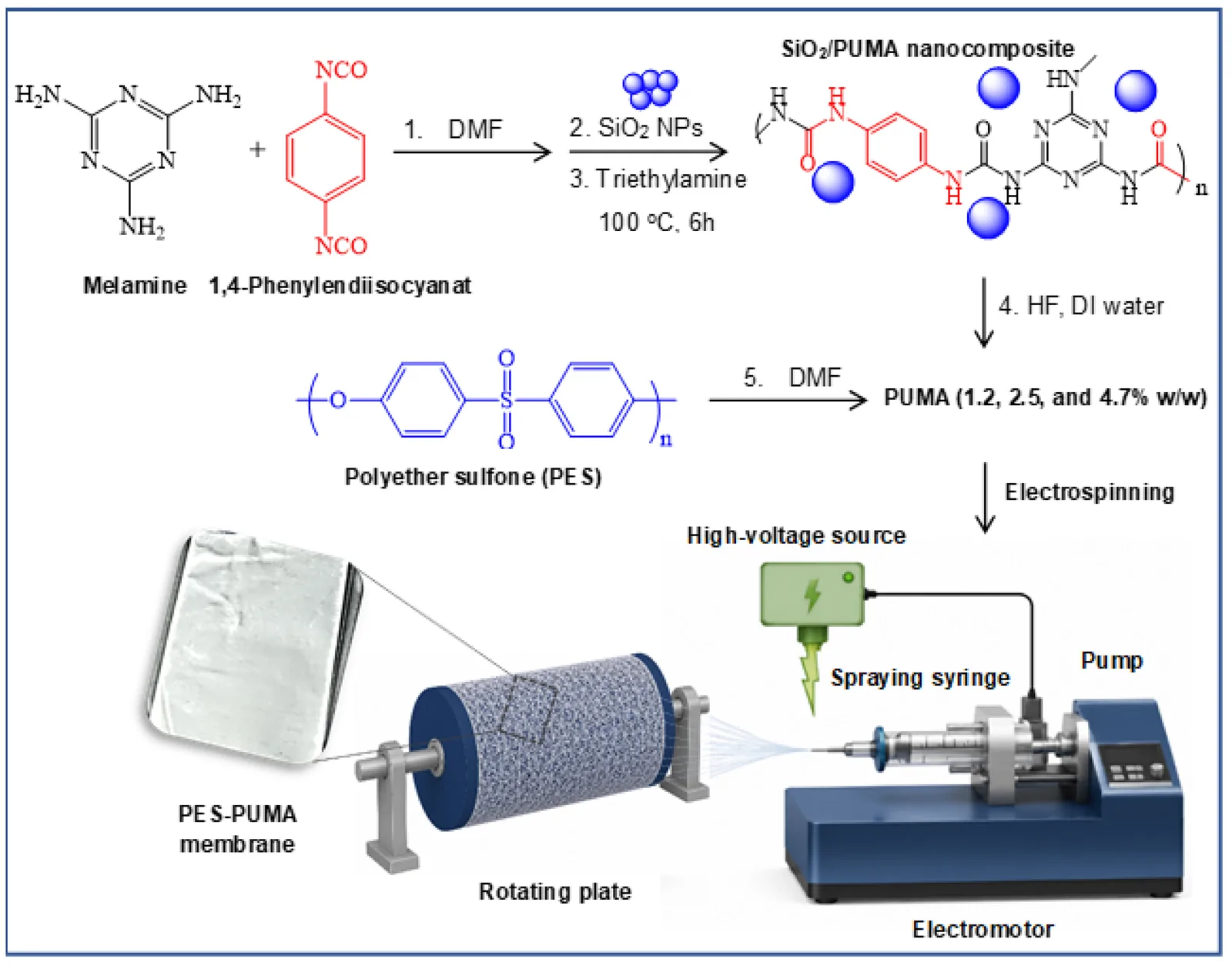
Fabrication strategy and synthesis pathway for the PES-PUMA composite membrane.

PES–PUMA membranes were fabricated by electrospinning homogeneous PES/PUMA solutions onto a rotating collector. PES was dissolved in DMF and subsequently mixed with PUMA to obtain nanocomposite solutions containing 1.2, 2.5, and 4.7 wt% PUMA, designated as PES–PUMA(1.2), PES–PUMA(2.5), and PES–PUMA(4.7), respectively. The principal fabrication route is illustrated in Scheme 1, while the electrospinning parameters and processing conditions are summarized in the Supplementary Material (Section S2.2, Table S2). As the synthesis and fabrication methodology was established in our previous study [12], only the essential procedures are summarized here, with emphasis in the present work placed on the effects of hydraulic operating conditions on membrane performance and fouling evolution.

### 2.3. Preparation of W/O Emulsion

The W/O emulsions were prepared using paraffin oil as the continuous phase and Span 80 as the nonionic surfactant, with deionized water as the dispersed phase. A water content of 10 vol% was selected to provide a consistent and sufficiently challenging feed for membrane evaluation. Sodium carbonate and sodium chloride were incorporated as stabilizing and ionic additives, respectively, and the resulting emulsions were homogenized under controlled conditions. The prepared emulsions exhibited no visible macroscopic phase separation during the experimental period. Detailed formulation conditions, the physicochemical properties of the paraffin oil, and rheological characteristics of the emulsions are provided in the Supplementary Material (Tables S3 and S4), consistent with the methodology established in our previous study [12].

### 2.4. Separation Experiments

Cross-flow filtration experiments were conducted at room temperature using the bench-scale setup previously described in our work [12], and its schematic configuration is therefore not reproduced here. Briefly, the PES–PUMA membrane was mounted in the filtration module with an effective area of 28.3 cm^2^ and a thickness of approximately 60–70 µm. Experiments were performed at room temperature under controlled hydraulic conditions, with operating pressures of 20–100 kPa and feed flow rates of 0.05–0.50 mL s^−1^. Oil permeate flux and water rejection were evaluated under each operating condition. The operating pressure was controlled directly using a peristaltic pump. Separate pressure measurements at the feed inlet and retentate outlet were not performed; therefore, the reported pressure values represent the pump-controlled operating pressure applied to the membrane system. The permeate side was maintained at atmospheric pressure.

For the cyclic filtration experiments, each cycle consisted of 30 min of continuous filtration, followed by the specified membrane cleaning procedure before the subsequent cycle. Ten consecutive cycles were performed, corresponding to a total filtration time of 300 min. Including the cumulative membrane-cleaning and transition periods, the total experimental duration was approximately 320 min. The experimental setup and operating principles were consistent with those reported previously [12], while the present study specifically examines the effects of hydraulic operating conditions on separation performance and fouling behavior.

Membrane separation experiments were performed under continuous cross-flow conditions for 30 min. The oil permeation flux, separation efficiency, flux-decline behavior and fouling parameters were evaluated using the procedures and equations provided in the Supplementary Material (Equations (S1)–(S6)). Membrane reusability was examined over ten successive filtration cycles. After each cycle, the proposed membranes were washed with *n*-hexane and dried at 50 °C for 1 h. Comprehensive descriptions of membrane and W/O emulsion characterizations, including governing equations for surface roughness and porosity, are provided in the Supplementary Materials (Equations (S7)–(S11)).

## 3. Results and Discussion

### 3.1. Characterization of PUMA Structure and PES-PUMA Membrane

#### 3.1.1. Chemical and Crystal Structure Study

**Figure 1:**
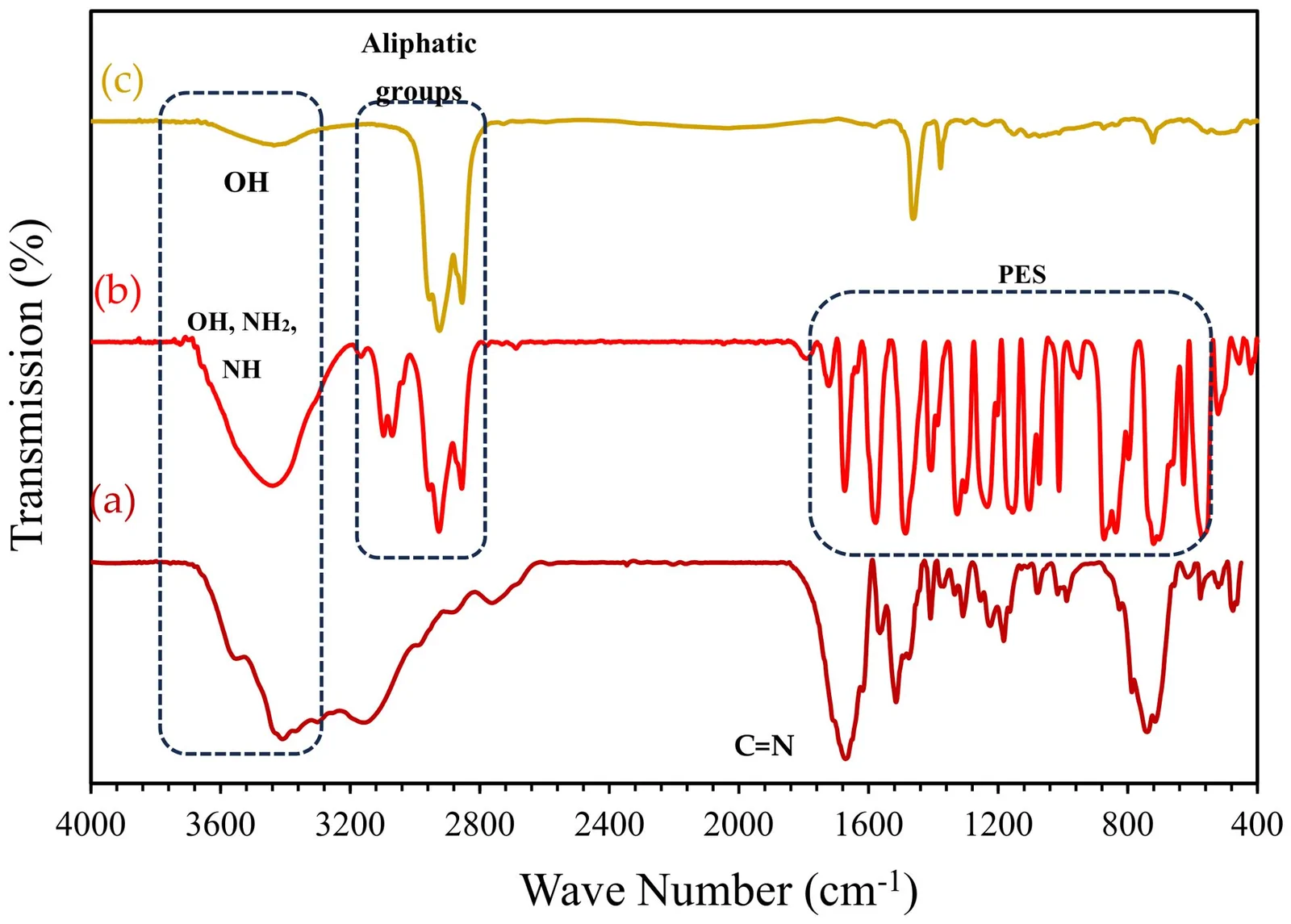
FT-IR spectra of (a) PUMA structure [12] (Permissions form: 261012-014096), (b) PES-PUMA(4.7) membrane after oil permeation, and (c) W/O emulsion (10% *v*/*v*).

The FT-IR spectra of the (a) PUMA structure, (b) PES-PUMA(4.7) after oil permeation, and (c) W/O emulsion (10% *v*/*v*) are presented in Figure 1. In the FT-IR spectrum of the PUMA structure (Figure 1a), broad absorption bands appearing in the range of 3300 and 3415 cm^−1^ correspond to stretching modes of the amine and secondary amine moieties. The sharp absorption peaks observed at 1634, 1669, and 1692 cm^−1^ are attributed to the C=N stretching vibrations of melamine aromatic rings. Furthermore, the disappearance of the absorption band at 1161 cm^−1^ demonstrates complete chemical leaching of the sacrificial silica nanoparticles [12]. The PES-PUMA(4.7) spectrum exhibits characteristic PES backbone vibrations, notably the S=O bands at 1151 and 1242 cm^−1^. The successful integration of PUMA into the PES matrix is confirmed by the distinct band at 1688 cm^−1^ [12].

After oil permeation, the FT-IR spectrum of the PES-PUMA highlights the interaction of the membrane structure with the W/O emulsion (10% *v*/*v*)—as shown in Figure 1b. The intense peaks at 2856–2939 cm^−1^ are characteristic of the aliphatic C-H stretching vibrations from the paraffin oil retained within the membrane structure. The bands at 442 cm^−1^ are attributed to the NH and NH_2_ stretching vibrations, while the peaks at around 3071–3097 cm^−1^ are assigned to aromatic C-H vibrations of PES matrix. The existence of characteristic peaks associated with the aliphatic groups of the W/O emulsion confirms successful oil permeation and retention within the membrane structure. In the FT-IR spectrum of the W/O emulsion, the broad band centered around 3409 cm^−1^ is attributed to water droplets dispersed within the contineous phase, while the peaks at 2856–2942 cm^−1^ correspond to the aliphatic groups of the paraffin oil (Figure 1c).

**Figure 2:**
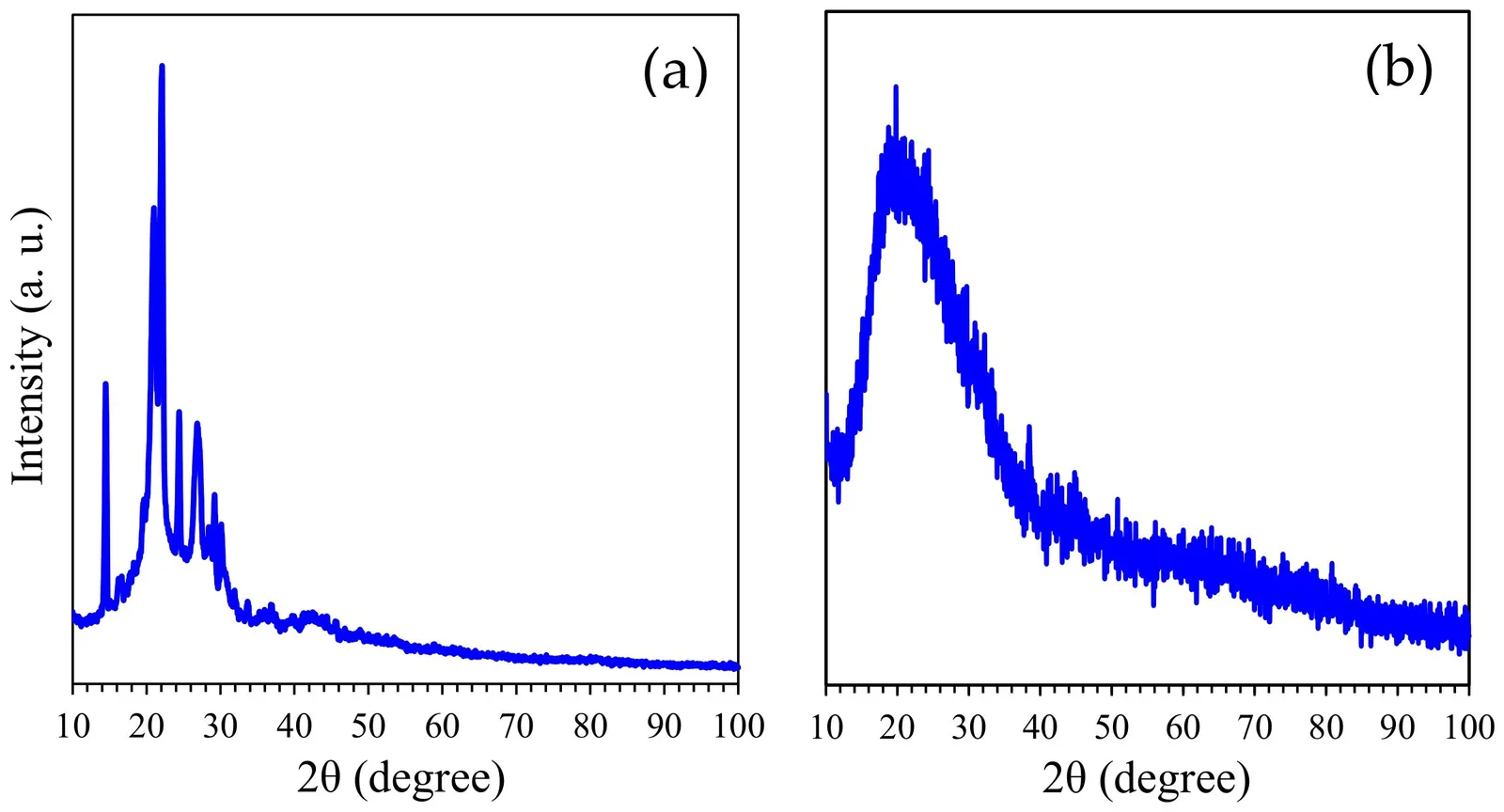
XRD spectra of (**a**) synthesized PUMA powder, and (**b**) PES-PUMA(4.7) membrane.

The crystallographic characteristics of the synthesized PUMA structure and the PES-PUMA(4.7) membrane are shown in Figure 2. The diffraction pattern represents the multiple overlapping phases or partially ordered PUMA structure. The XRD pattern of the PUMA structure exhibits a semi-crystalline nature, characterized by the combination of broad and sharp diffraction peaks (Figure 2a). The sharp and well-defined peaks in the 2θ range of 15–32° are attributed to the semi-crystalline domains, while the broad peaks beyond roughly 35° suggest an amorphous or less ordered phase of the PUMA structure, which may be attributed to the disordered arrangement of long-chain segments in the polymer material. The broad diffraction features observed in the 2θ range of 25–29° can be attributed to the characteristic scattering of MA functional groups. This result is in good agreement with the XRD patterns of the MA (2θ: 21.74°, 22.14°, 26.28°, 26.51°, 28.90°, 29.89° and 35.91°) [24] and PU (2θ: 19.0°, 21.35°, and 43.0°) [25]. Furthermore, the absence of detectable silica-related signals in the XRD pattern at 2θ = 36.5°, 39.5°, 40.3°, 42.5°, 45.8°, and 56.9° [26] indicates that no appreciable crystalline or vibrationally detectable silica phase remained after the template-removal treatment.

The XRD pattern of the PES-PUMA(4.7) membrane shows a broad diffraction peak located at 2θ of 18.1° (Figure 2b), which is characteristic of the amorphous nature of PES matrix [27]. Compared with the pure PUMA structure, the disappearance of the sharp crystalline reflections suggests that the crystalline PUMA structure is effectively dispersed within the amorphous PES matrix. These results indicate a high degree of compatibility between the PUMA structure and the PES matrix. This high degree of compatibility makes the membrane suitable for maintaining both structural integrity and stable permeability during the filtration process.

#### 3.1.2. Surface Morphology Study

**Figure 3:**
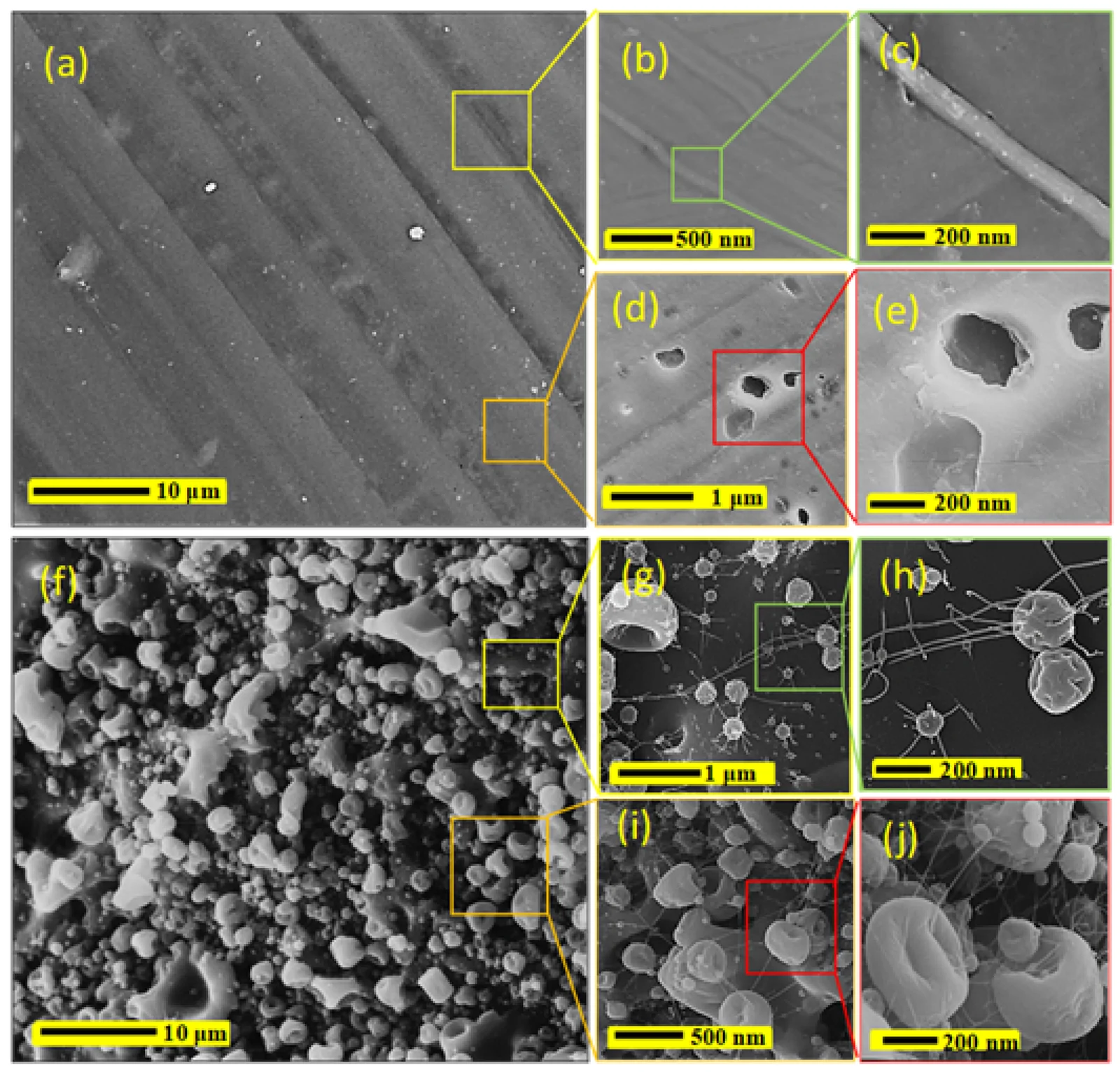
FE-SEM images of (**a**–**e**) PES and (**f**–**j**) PES-PUMA(4.7) membranes under low- and high-magnification views (from left to right, respectively).

The surface morphology of the (a–e) PES and (f–j) PES-PUMA(4.7) membranes was studied by FE-SEM analysis, and the results are shown in Figure 3. Low-and high-magnification images are presented to provide an overview of the overall morphology and surface details, respectively. The low-magnification SEM image of the PES membrane exhibits a homogeneous and smooth surface without visible macroscopic defects, cracks, or delamination, indicating good membrane structural integrity (Figure 3a). The presence of PES nanofibers with diameters of approximately 400 nm, extending to micrometer-scale lengths, is clearly visible on the membrane surface (Figure 3b,c). In addition, the membrane surface is predominantly populated with irregularly shaped pores, exhibiting an average pore diameter of approximately 380 nm (Figure 3d,e).

The SEM image of the PES-PUMA(4.7) membrane revealed a highly rough and heterogeneous surface composed of aggregated nodules, bead-like domains, and interconnected fibrous features, indicating that the incorporation of PUMA substantially modified the membrane microstructure and promoted the formation of a hierarchical surface morphology (Figure 3f–h). High-magnification images confirmed the presence of localized, hollow, and donut-shaped particles on the PES-PUMA(4.7) membrane (Figure 3i,j). In our previous study [12], the membrane surface was only described as an inhomogeneous structure with partially aggregated features. However, the present images show new structural details of the heterogeneous membrane surface.

The SEM images of the PES-PUMA(1.2 and 2.5) membranes are presented in the Supplementary Materials (Figure S2). These images show that increasing the PUMA content from 1.2% to 2.5% causes a marked alterations in the surface morphology. The density of irregular structures progressively increases with a higher PUMA content, reaching its maximum in the PES-PUMA(4.7) membrane. The presence of discrete particles and interconnected electrospun nanofibers suggests a complex micro/nanostructure rather than a simple, smooth surface layer. This structure may be caused by phase separation and partial polymer aggregation during rapid solvent evaporation and solidification [28,29]. The wettability characteristics of the PES–PUMA(4.7) membrane were previously evaluated using water contact angle measurements and oil drop penetration time tests on the membrane surface [12]. The reported wettability profile indicates preferential affinity toward the oil phase while limiting water spreading on the membrane surface, thereby supporting selective oil transport and water rejection during W/O emulsion separation.

**Figure 4:**
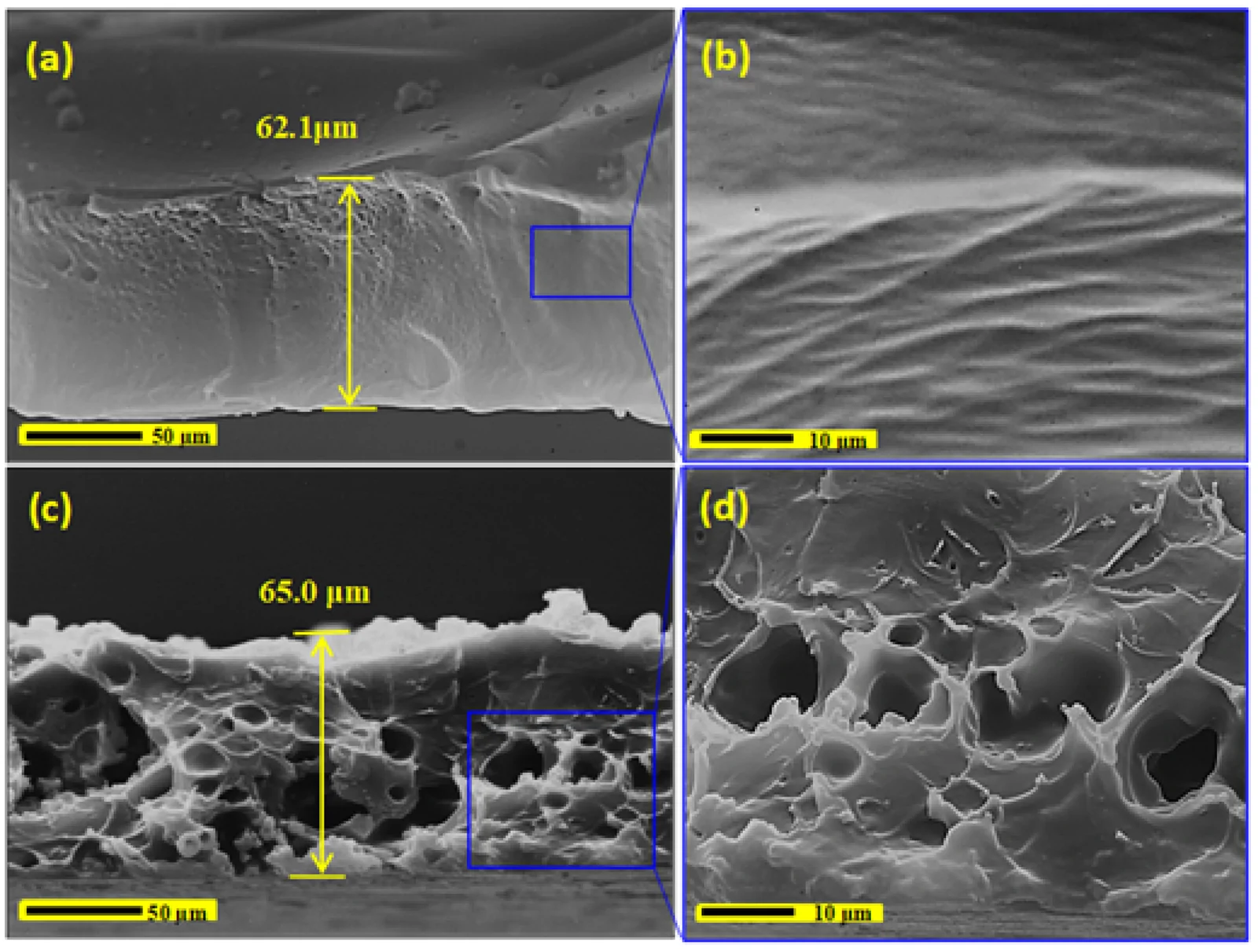
The FE-SEM cross-sectional images of (**a**,**b**) PES and (**c**,**d**) PES-PUMA(4.7) membranes under low magnification (**a**,**c**) and high magnification (**b**,**d**).

The cross-sectional SEM images of the (a and b) PES and (c and d) PES-PUMA(4.7) membranes are shown in Figure 4. Indexes “a” and “c” represent low-magnification images, while “b” and “d” denote high-magnification images. Depending on the incorporation of PUMA into the structure of the PES membrane, the thickness of the resulting films varies within 65 ± 7 μm. The surface structure of the resulting membrane is composed of a well-defined, multilayered porous structure associated with interconnected nanofibrous networks and submicron pores formed by the PES matrix (Figure 4a,c). The formation of a hierarchical framework with abundant hollow structures is linked to numerous interaction sites and continuous channels (Figure 4b,d). These structures provide effective pathways for fluid transport through the layered porous network. Therefore, the mesoporous structure and the hydrophobic nature of PUMA lead to increased oil permeability across the PES–PUMA(4.7) membrane.

FE-SEM cross-sectional images of the PES-PUMA(1.2) and PES-PUMA(2.5) membranes are provided in the Supplementary Materials (Figure S3). The structure of the PES membrane was markedly altered as the PUMA content in the PES matrix increased. The incorporation of higher PUMA concentrations promoted the formation of a greater number of pores and interconnected hollow channels, resulting in a porous structure. This morphological evolution is expected to facilitate fluid transport and improve membrane permeability by reducing hydraulic resistance and enhancing pore connectivity.

#### 3.1.3. Thermal Stability Study

**Figure 5:**
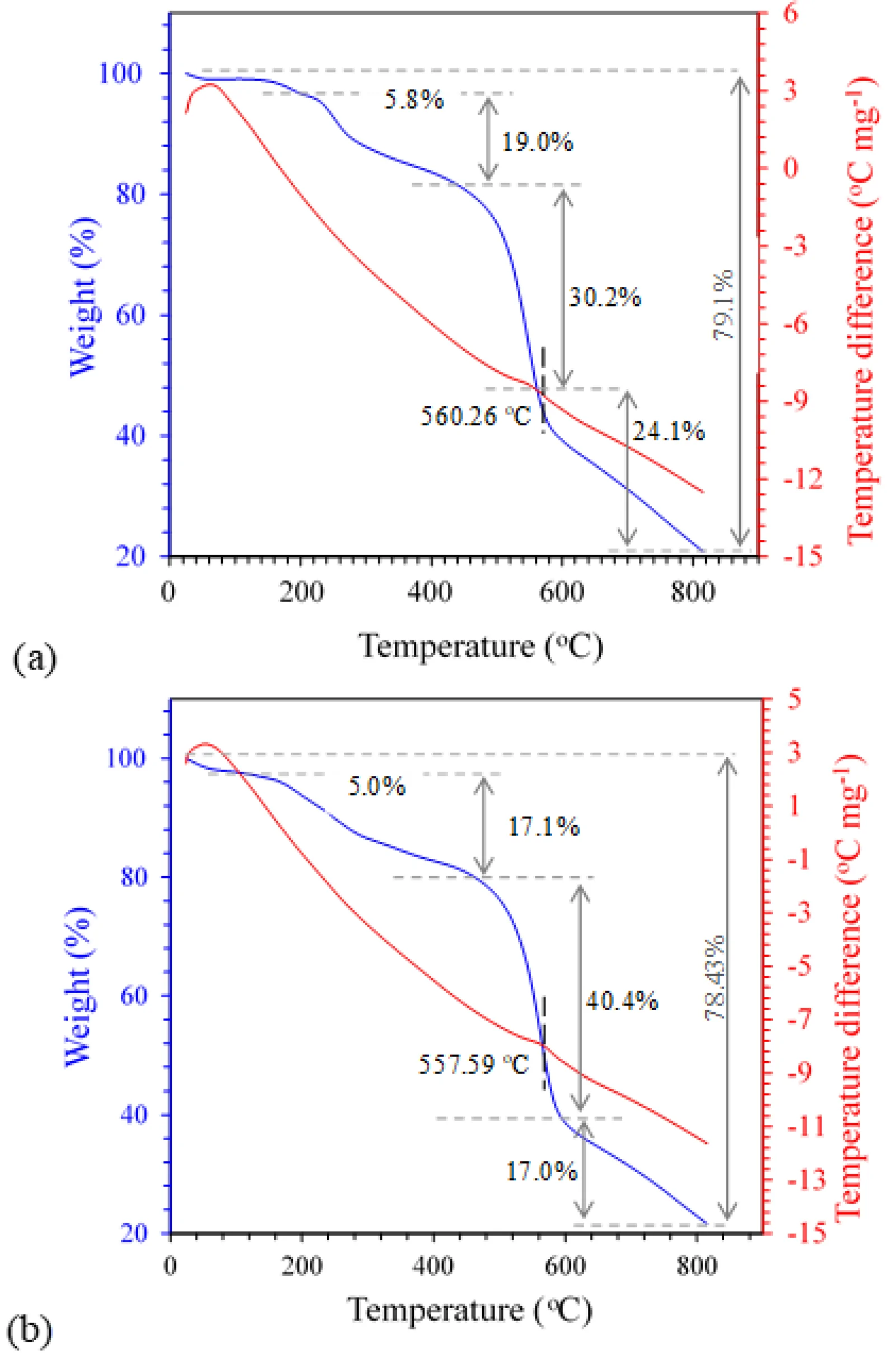
TGA-DTA curves of (**a**) PES membrane and (**b**) PUMA structure.

The thermogravimetric behavior of the (a) PES membrane and (b) PUMA structure is shown in Figure 5. The TGA curves indicate that the samples undergo multi-step thermal decomposition. The PES membrane exhibits a cumulative weight loss of 79.1%, primarily attributed to solvent evaporation and the thermal decomposition of the PES matrix (Figure 5a). An initial weight loss of approximately 5.8% was observed in the temperature range of 30–244 °C. This phase is mainly associated with the desorption of physically adsorbed molecules and the removal of residual DMF solvent trapped within the membrane pores. These findings are in good agreement with those of the FT-IR analyses. The thermal degradation of the polymeric framework then proceeds through three distinct stages. The first decomposition stage was observed between 277 and 517 °C, yielding a weight loss of approximately 19.0% due to the initial polymer degradation. The second and most significant degradation stage occurred between 517 and 586 °C, with a mass loss of 30.2% and an inflection temperature at 560.26 °C, corresponding to the main cleavage of the ether and sulfone linkages. The final stage (586 to 800 °C), with a 24.1% weight loss, was related to the slow pyrolysis of the residues.

The PUMA structure exhibited a multi-step decomposition profile, beginning with an initial weight loss of 5% between 25 and 200 °C due to the evaporation of the physically adsorbed DMF solvent (Figure 5b). Subsequently, the polymer underwent three distinct degradation stages. The first stage, occurring between 200 and 450 °C, showed a 17.1% mass loss corresponding to the cleavage of urethane and urea linkages. The second stage occurred between 450 and 600 °C, with a significant mass loss of 40.4% and a derivative maximum at 557.59 °C, reflecting the high thermal resistance of the aromatic backbone and melamine rings. The final pyrolytic decomposition stage (600–800 °C) resulted in a cumulative loss of 78.43%, leaving a 21.57% solid residue at 800 °C. This high thermal stability (557.59 °C) is primarily attributed to the restricted segmental mobility within the cross-linked network.

The thermal degradation behavior of the PES–PUMA(4.7) membrane was previously characterized and reported in our earlier study [12]. The corresponding TGA profile showed a total weight loss of 98.9%, with an initial weight loss of approximately 5.0% at 30–240 °C, followed by additional degradation stages at higher temperatures. The main degradation stage occurred at approximately 443.50 °C, with a weight loss of 41.7%. The previously reported results also showed that the PES–PUMA(4.7) membrane exhibited higher thermal stability than the PUMA structure, which was attributed to the rigid aromatic structure of PES and the restricted segmental mobility of the PUMA structure.

The thermal stability follows the order PES > PES–PUMA > PUMA, which agrees with the structural interactions observed in the TGA curves. The thermal shift and single-peak behavior suggest interfacial compatibility and molecular interactions between the PES matrix and the PUMA structure. These findings are in good agreement with those of the FT-IR and XRD analyses.

**Figure 6:**
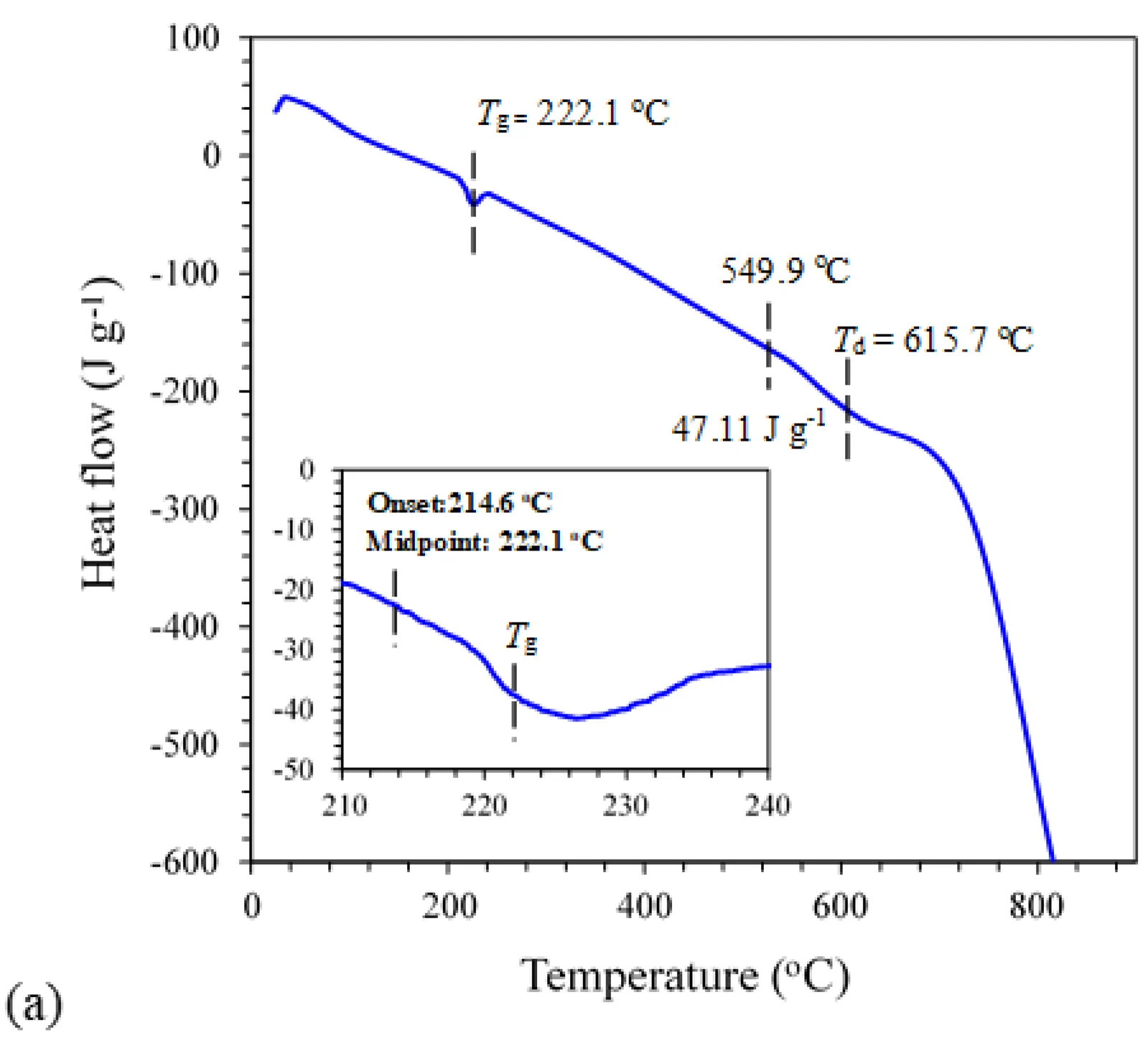

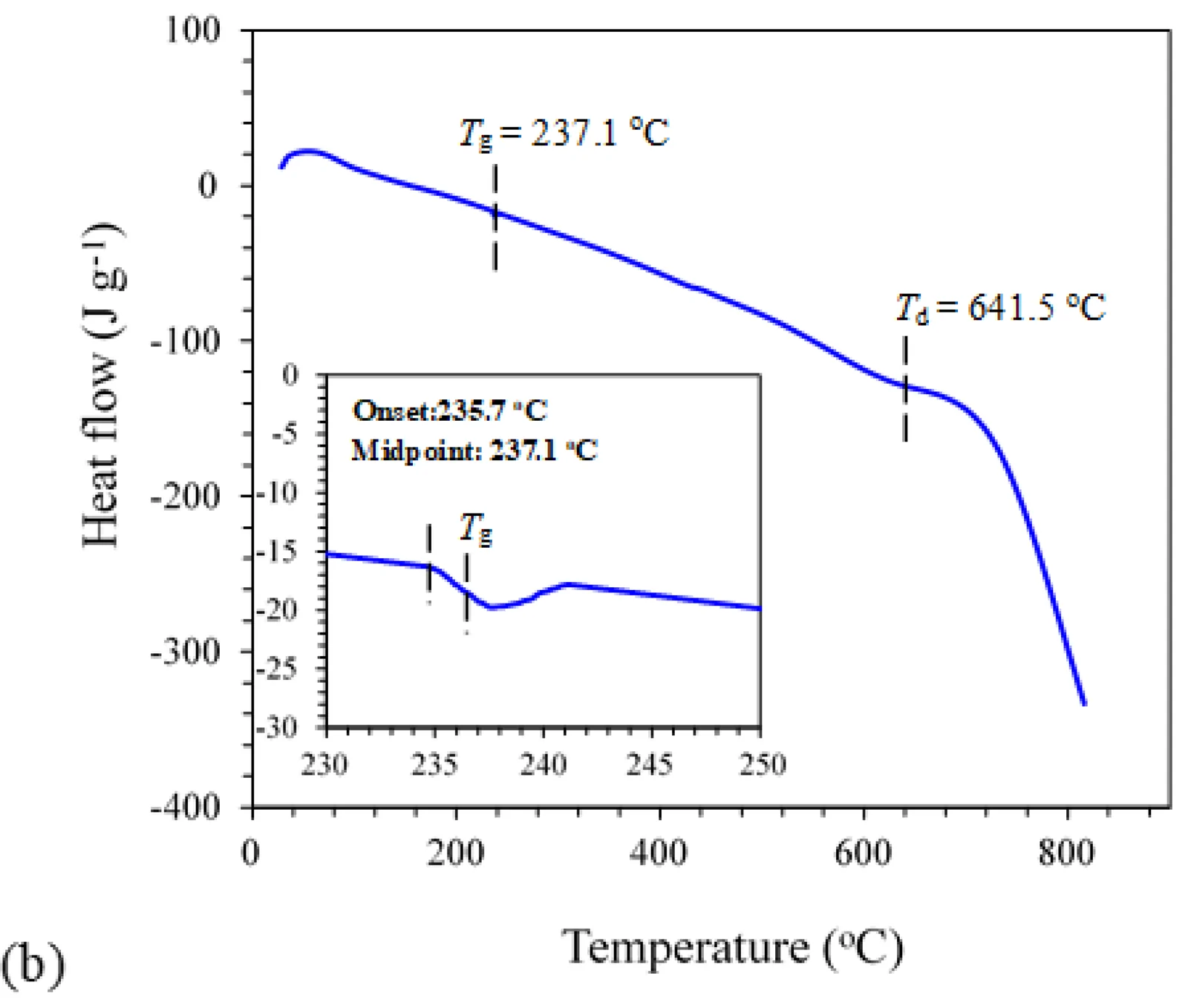

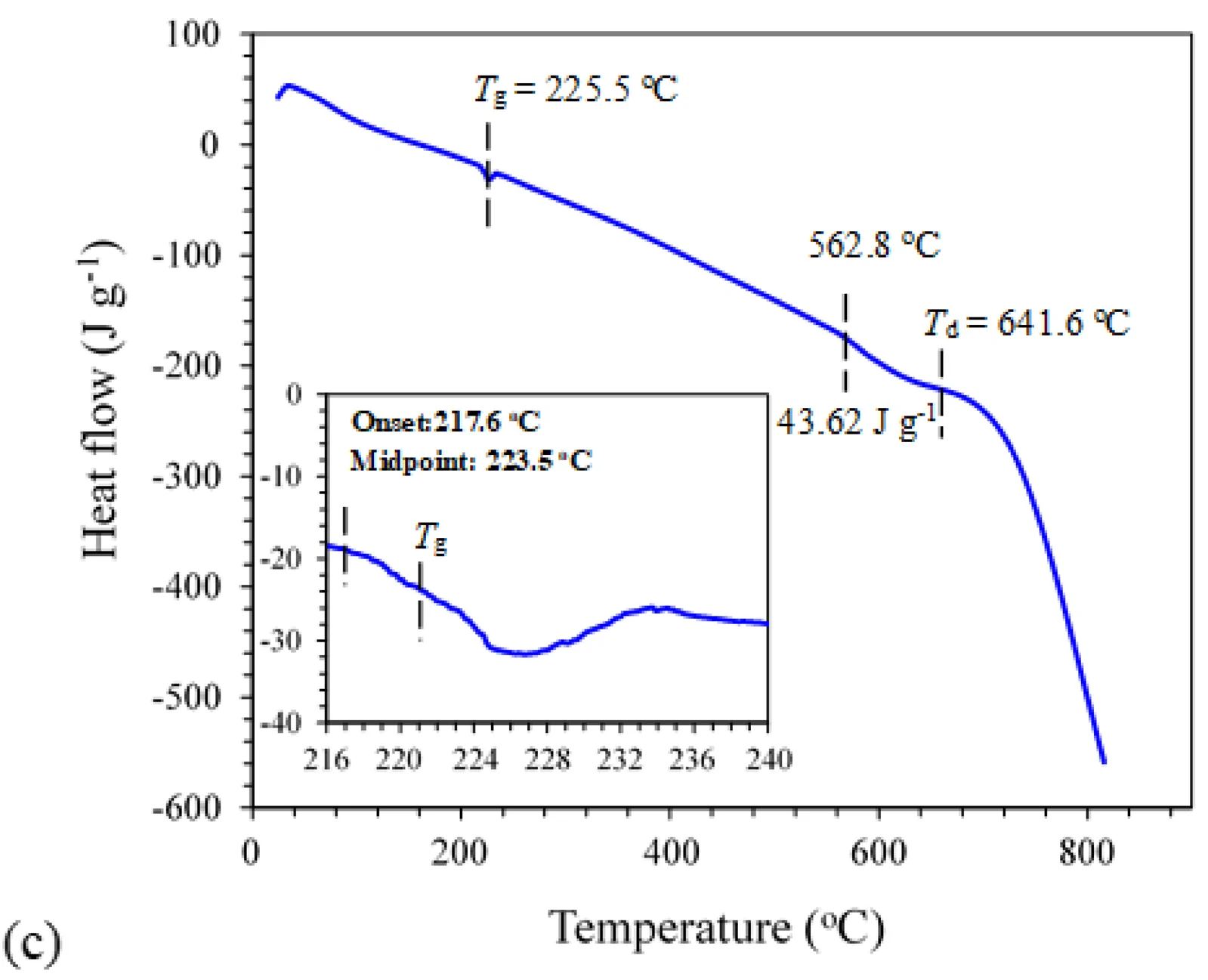
The DSC curves of the (**a**) PES membrane, (**b**) PUMA structure, and (**c**) PES-PUMA(4.7) membrane.

The thermal characteristics of the (a) PES membrane, (b) PUMA structure, and (c) PES-PUMA(4.7) membrane were evaluated by DSC analysis at a heating rate of 20 °C min^−1^, and the results are shown in Figure 6. No significant differences were observed among the three samples in the low-temperature region of 25–60 °C. This behavior may be associated with the limited amount of residual moisture or solvent in the samples. The PES membrane, PUMA structure, and PES–PUMA(4.7) membrane exhibited distinct glass transition temperatures (*T*_g_) at 222.1 °C, 237.1 °C, and 225.5 °C, respectively. The difference in *T*_g_ between the PES and PES–PUMA(4.7) membranes suggests that the incorporation of the PUMA structure affects the chain mobility and the thermal response of the membrane. In general, a decrease in cross-link density shifts the glass transition to lower temperatures, while the plasticizing effect of the PES matrix can further enhance the segmental motion of the polymer chains.

The elevated *T*_g_ (225.5 °C) indicates a highly stable and rigid polymer network, ensuring that the membrane remains in the glassy state throughout the experimental procedures. This minimizes the risk of thermally induced structural deformation or relaxation during chemical cleaning cycles. While this thermal robustness contributes to the membrane’s overall stability, the direct impact of *T*_g_ on long-term mechanical fatigue during cycling warrants further investigation.

In addition, the presence of a single *T*_g_ for the PES–PUMA(4.7) membrane is consistent with good interfacial compatibility between the PUMA structure and the PES matrix, suggesting possible intermolecular interactions between the two components. The PES membrane exhibited two thermal degradation stages at 517.08–549.93 °C and 615.35–725.22 °C, with enthalpies of 47.11 J g^−1^ and 50.94 J g^−1^, respectively. The PUMA structure showed thermal degradation at 523–563 °C and 641.6–730.7 °C, with enthalpies of 43.62 J g^−1^ and 35.63 J g^−1^, respectively. The PES-PUMA(4.7) membrane showed a predominant single decomposition stage at 613.45–725.01 °C, with an enthalpy of 28.73 J g^−1^. The shift in the degradation behavior and the reduced enthalpy of the PES–PUMA(4.7) membrane indicate that incorporation of the PUMA structure modifies the thermal response of the PES matrix. These changes may be associated with intermolecular interactions between PES and PUMA, including hydrogen bonding and π–π stacking, as supported by FTIR analysis [12].

### 3.2. Characterization of Membrane After Separation Cycle

#### 3.2.1. Pore Size Distribution and Porosity Determination

**Figure 7:**
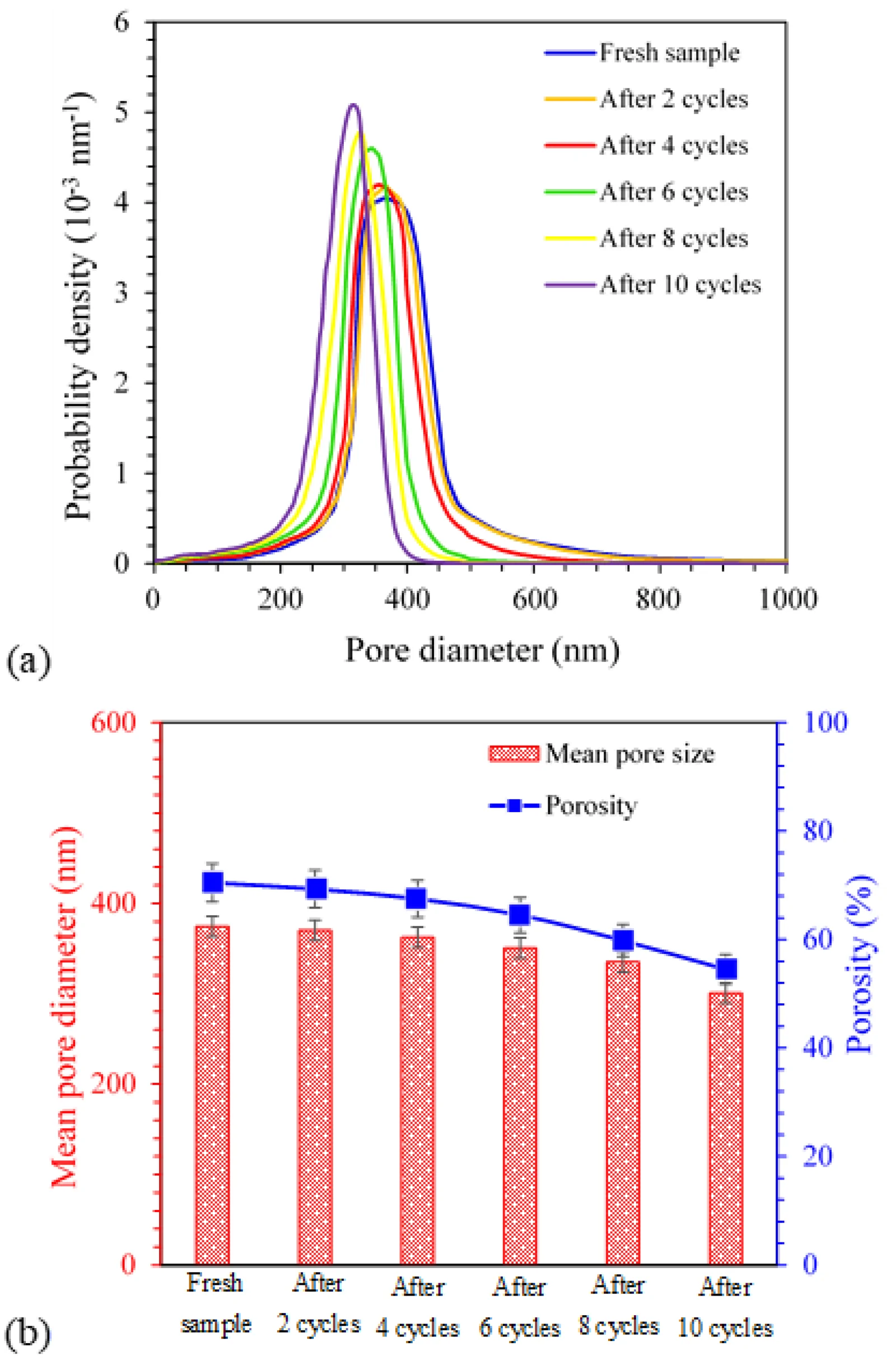
(**a**) The pore size distribution and (**b**) mean pore diameter and porosity of the PES-PUMA(4.7) membrane after different separation cycles with cleaning treatment operated at 60 kPa [data are presented as mean ± SD (*n* = 3)].

The mean pore size, pore size distribution, and porosity of the PES–PUMA(4.7) membrane after different filtration and cleaning cycles are presented in Figure 7. As shown in Figure 7a, the pore size distribution of the PES-PUMA(4.7) membrane ranges from approximately 50 to 1000 nm, consistent with our previous study. After ten cycles, the membrane exhibited a narrower pore size distribution (~20–420 nm) and the main peak shifted from 360 to 310 nm, indicating pore constriction caused by foulant deposition and preferential blockage of larger pores (>500 nm). These changes suggest the formation of a cake layer on the membrane surface during separation cycles.

The mean pore size and porosity of the PES–PUMA(4.7) membrane decreased as the number of cycles increased (Figure 7b). The mean pore sizes after two and ten cycles were 387 nm and 278 nm, respectively, with corresponding porosities of 70 ± 6.0% and 54 ± 5.0%. These results indicate that the pore size range of interfiber voids within the PES-PUMA(4.7) membrane decreases due to the accumulation of retained emulsion components, leading to local structural inhomogeneity and pore blockage. Although the changes after two cycles were minor, a significant decline in porosity was observed after ten cycles. This reduced porosity restricts the permeation of the continuous oil phase. The narrowed pore size distribution and reduced mean pore size may contribute to increased resistance to water-droplet passage, whereas the loss of porosity may adversely affect long-term oil permeate flux.

At an operating pressure of 60 kPa, the mean pore size decreased from approximately 374 nm to 300 nm (a reduction of about 19.8%), while the porosity declined from approximately 70% to 54% (a decrease of about 22.9%) after operation. These structural changes likely result from the combined effects of pressure-induced compaction and fouling-related pore blockage or foulant deposition. The observed increase in foulant-layer thickness with rising feed flow rate further supports the role of foulant deposition in these structural changes. However, the current experiments do not allow for a quantitative separation of the individual contributions of mechanical compaction and fouling. A control experiment using pure oil at the same operating pressure (60 kPa) would be necessary to isolate the pressure-induced structural effects from those caused by emulsion-induced fouling and pore blockage.

#### 3.2.2. Topography and Surface Roughness Study

**Figure 8:**
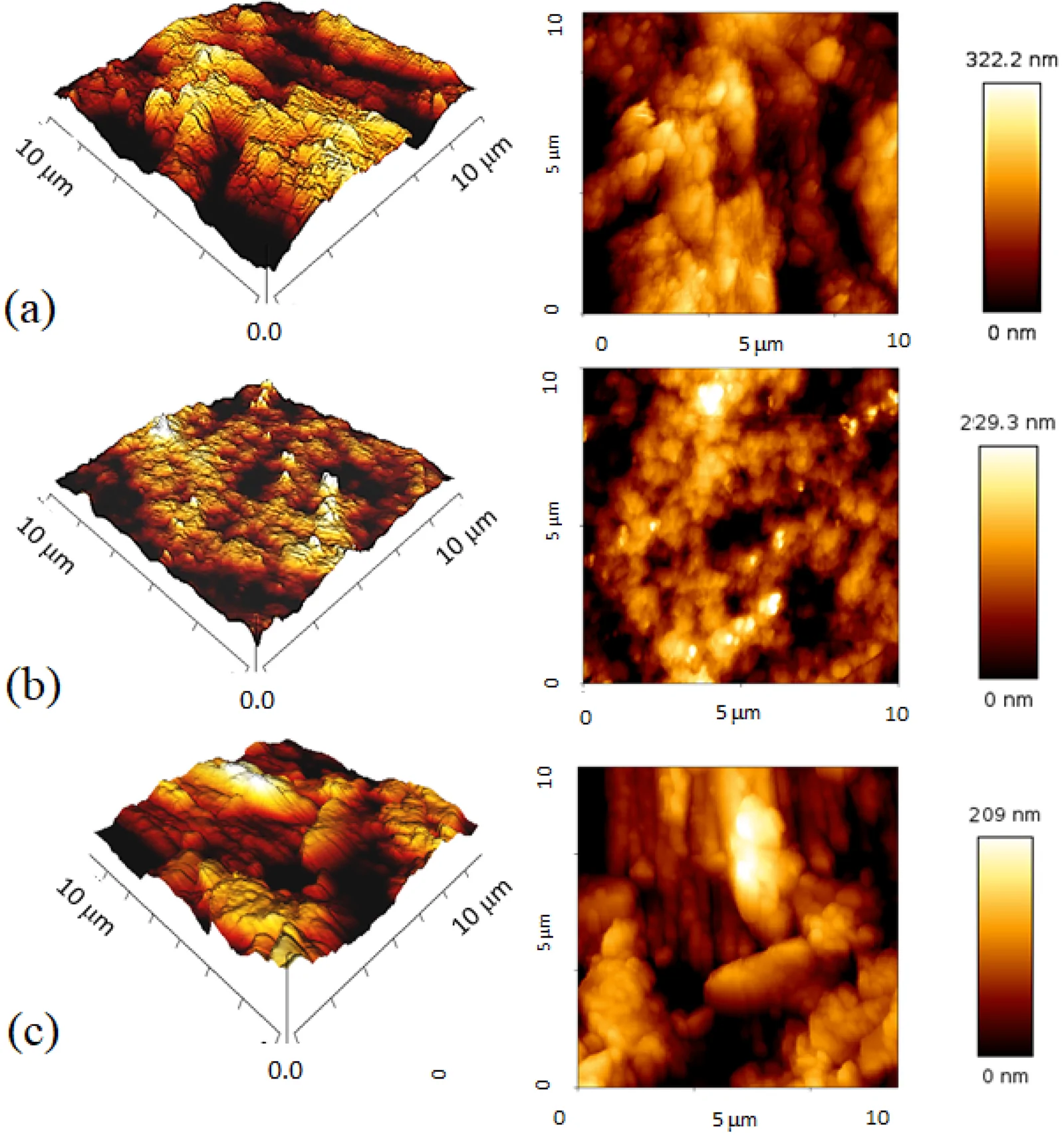

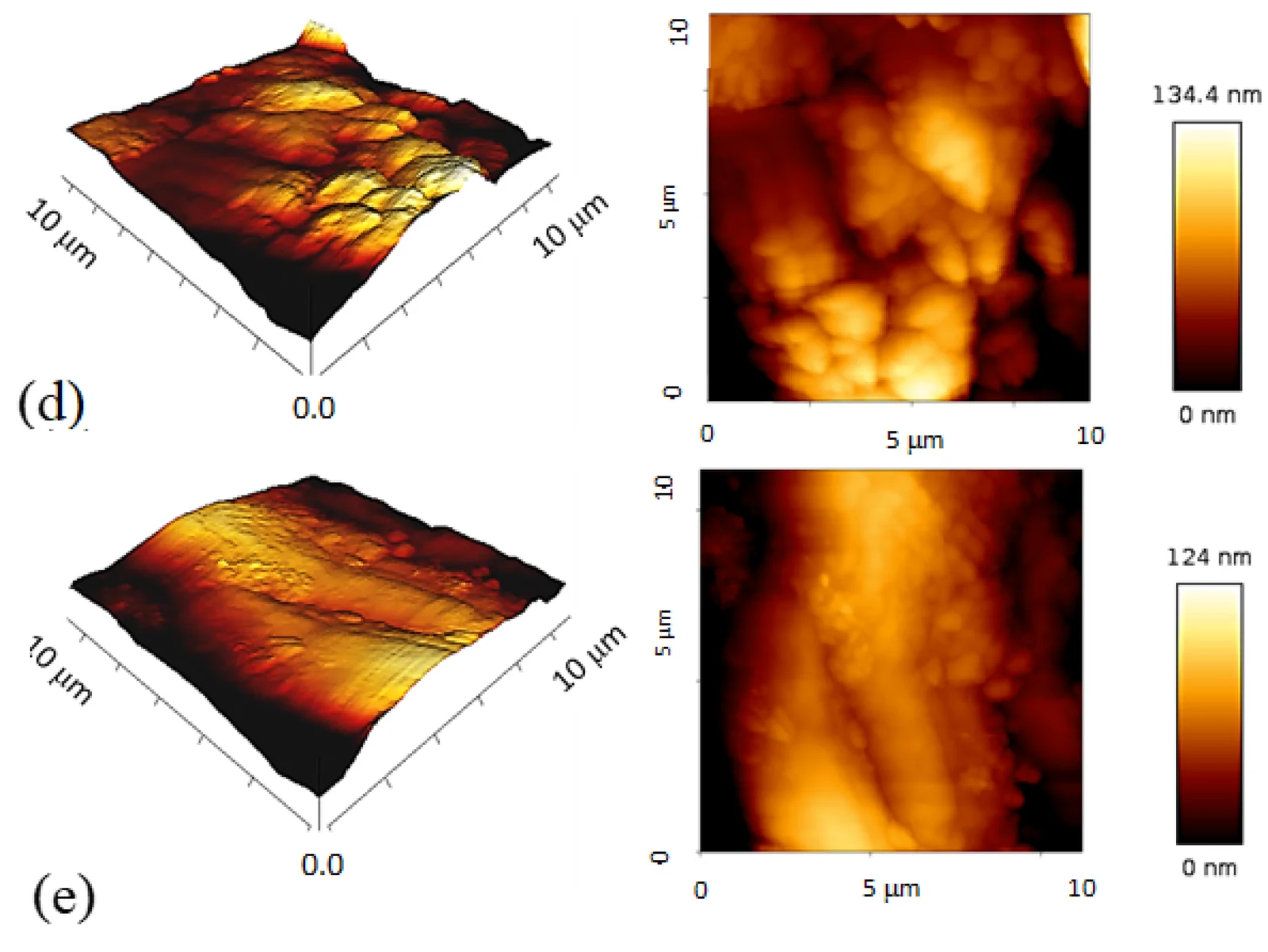
The AFM (3D and 2D) images of the PES-PUMA(4.7) membrane obtained from different separation cycles (**a**): 2 cycles, (**b**) 4 cycles, (**c**) 6 cycles, (**d**) 8 cycles, and (**e**) 10 cycles.

The AFM images of the PES–PUMA(4.7) membrane after successive filtration cycles followed by cleaning are presented in Figure 8, including the recycled membranes after (a) 2, (b) 4, (c) 6, (d) 8, and (e) 10 cycles. The roughened structure can markedly enhance the surface wettability, imparting excellent oleophilicity and hydrophobicity. The surface morphology and roughness characteristics of the fresh PES–PUMA(4.7) membrane have been reported in our previous study [12].

The 3D and 2D AFM images reveal significant changes in membrane surface morphology after separation cycles. The recycled membranes exhibited alterations in surface topography, consistent with surface modifications induced by fouling and subsequent cleaning. The observed changes in surface depth range suggest a reduction in surface roughness, which can be attributed primarily to (i) pore blocking and valley filling and (ii) the formation of a foulant-covered layer. Thus, the membrane surface changes from a rough morphology to a relatively smoother, foulant-covered topography as separation progresses [30].

The dark grooves visible in the AFM images indicate localized surface non-uniformities. The surface roughness of proposed membrane depends on the interplay between the characteristic size and morphology of the surface features and the size distribution of foulant particles and their aggregates. The deepest grooves in the PES–PUMA(4.7) membrane after two cycles reached 322.2 nm, which is significantly smaller than the membrane thickness (60 ± 7 μm).

The interaction between the W/O emulsion and the PES-PUMA(4.7) membrane during the separation process can significantly alter the surface topography through multiple mechanisms, including oil adsorption, pore blockage, residual surfactant accumulation, deposition of fine impurities, and the formation of a heterogeneous fouling layer. It is important to note that membrane roughness is influenced by several factors, such as emulsion stability, droplet size distribution, surfactant concentration, the number of separation cycles, transmembrane hydraulic pressure, surface wetting, and the cleaning protocol [31,32]. The transition to a relatively smooth, foulant-covered morphology is associated with a progressive decline in oil permeate flux during W/O emulsion separation. However, the effect of surface roughness on oil permeate flux ultimately depends on the membrane structure and operating conditions [33].

Surface roughness plays a crucial role in modulating membrane fouling by influencing interfacial properties such as the contact area, the wetting state, and the accessibility of surface defects [34]. An increase in surface roughness can increase the effective surface area of the membrane and accelerate fouling [35,36]. Nevertheless, our results show that the separation operation can itself modify the membrane surface morphology and roughness over repeated cycles, which subsequently affects separation [37].

The surface roughness parameters of the PES–PUMA(4.7) membrane, obtained from AFM analysis after various separation and cleaning cycles, are presented in Table 1. For each cycle condition, multiple distinct AFM scan lines/areas were analyzed, and the roughness parameters are reported as means ± standard deviations (SDs). These measurements represent spatial replicates of the same membrane and account for local surface heterogeneity and measurement variability.

Based on the results, a consistent reduction in all roughness parameters including *R_p_*, *R_v_*, *R_a,_* and *R_q_* is observed as the number of separation cycles increases. Notably, *R_p_* decreased from 235.65 nm for the fresh sample to 88.51 nm after ten cycles, while *R_v_* decreased from 129.35 nm to 35.49 nm. The decrease in peak heights and valley depths provides quantitative evidence for the “valley filling” effect, wherein deposited foulants, residual surfactants, and water droplets accumulate within the surface voids.

A comparison of these results confirms a significant smoothing of the membrane surface compared to the fresh sample, as indicated by the substantial decline in *R_a_* (from 26.11 nm to 6.48 nm) and *R_q_* (from 29.83 nm to 8.65 nm) values. The rough hydrophobic surface of the fresh membrane initially promotes a Cassie–Baxter wetting state by trapping air pockets within surface asperities, which reduces the solid–liquid contact area [38]. However, the accumulation of foulants within surface voids and the formation of a uniform cake layer on the membrane structure lead to reduced surface roughness. This correlation between decreasing *R_a_* values and declining oil permeate flux, suggests that the initial surface irregularity enhances the capture of emulsified droplets, thereby accelerating pore blockage and cake layer formation.

### 3.3. Characterization of W/O Emulsion

#### Droplet Size Distribution

**Figure 9:**
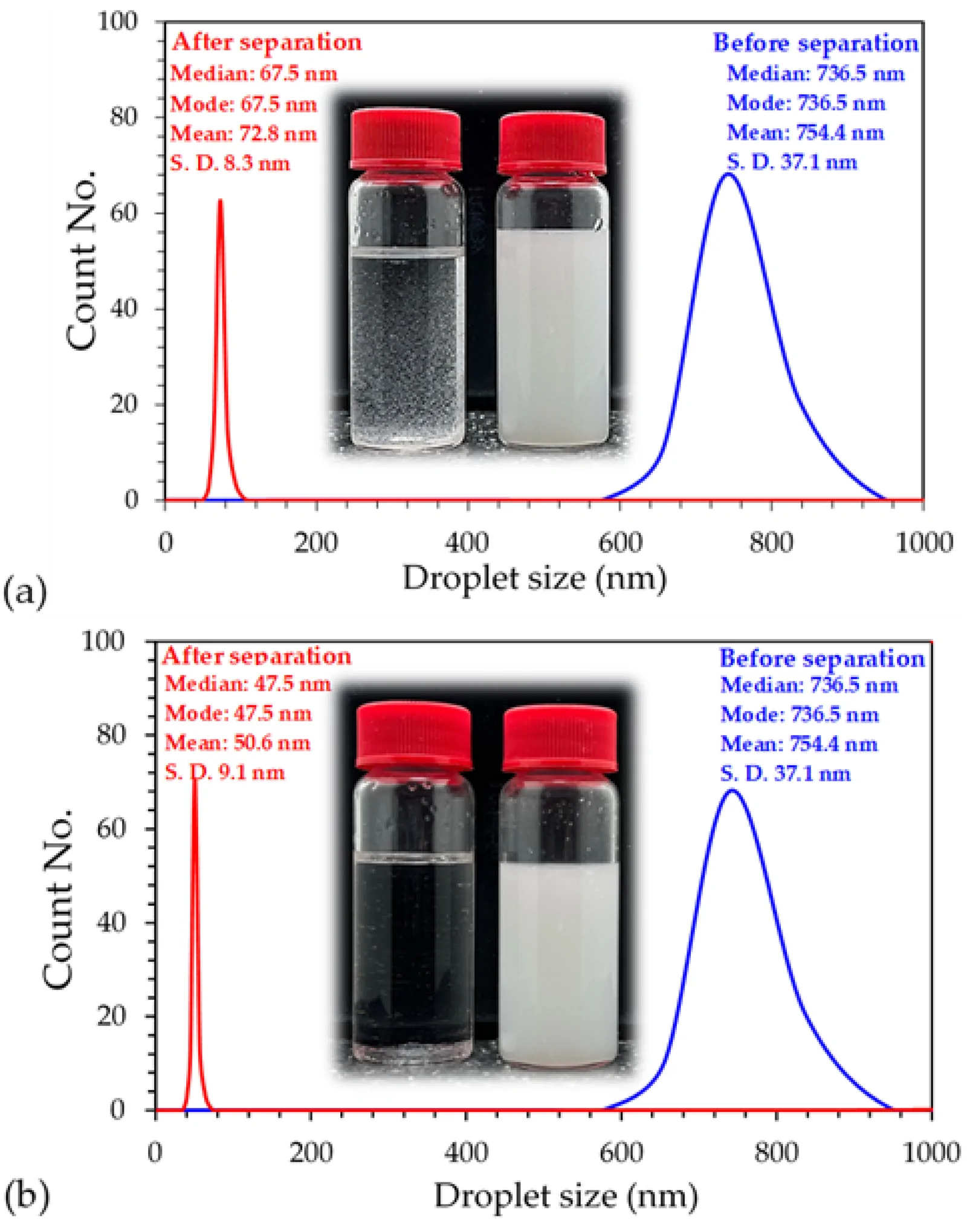
The droplet size distribution of W/O emulsions (10% *v*/*v*) before and after separation by the (**a**) PES and (**b**) PES-PUMA(4.7) membranes.

The droplet size distribution of the W/O emulsion (10% *v*/*v* water) before and after separation was analyzed by dynamic light scattering (DLS) (Figure 9). The primary emulsion exhibited a broad size distribution, ranging from 580 to 948 nm, with a mean diameter of 754 ± 37 nm (Figure 9a). The mean pore diameters of the PES membrane (468 ± 21 nm) and the PES-PUMA(4.7) membrane (374 ± 27 nm) were smaller than the average droplet size of the W/O emulsion. Although the mean diameter of the emulsion droplets (754 nm) was larger than the mean diameter of the membrane pores (374 nm), the droplet-to-pore size ratio alone is not sufficient to describe their transport behavior during filtration. At high operating pressures, water droplets approaching the membrane pores from the feed side may undergo pressure-induced deformation at the pore entrances. This pressure-dependent deformation can weaken the size-sieving contribution and contribute to the observed decrease in water rejection from 93.1 ± 2.1% at 20 kPa to 78.2 ± 1.8% at 100 kPa. In addition, pressure-induced pore constriction and foulant deposition can modify the effective transport pathways, further influencing the pressure-dependent separation behavior.

The separation process is likely attributed to a synergistic combination of size-sieving, coalescence, and demulsification effects [39,40]. After separation, a significant reduction in both the range and average droplet size was observed for the filtrates. The mean droplet size of the filtrate decreased from 754.4 nm ± 24.6 to 72.8 ± 8.3 nm for the PES membrane and to 50.6 ± 9.1 nm for the PES-PUMA(4.7) membrane. The permeate of the PES membrane indicated the presence of smaller water droplets (Figure 9a). The PES-PUMA(4.7) membrane yielded a highly transparent permeate (Figure 9b). These results indicate the high potential of the PES-PUMA(4.7) membrane’s separation efficiency and enhanced demulsification performance compared to those of the PES membrane.

### 3.4. Operating Parameters of Membrane Performance

#### 3.4.1. Operating Pressure Effect

**Figure 10:**
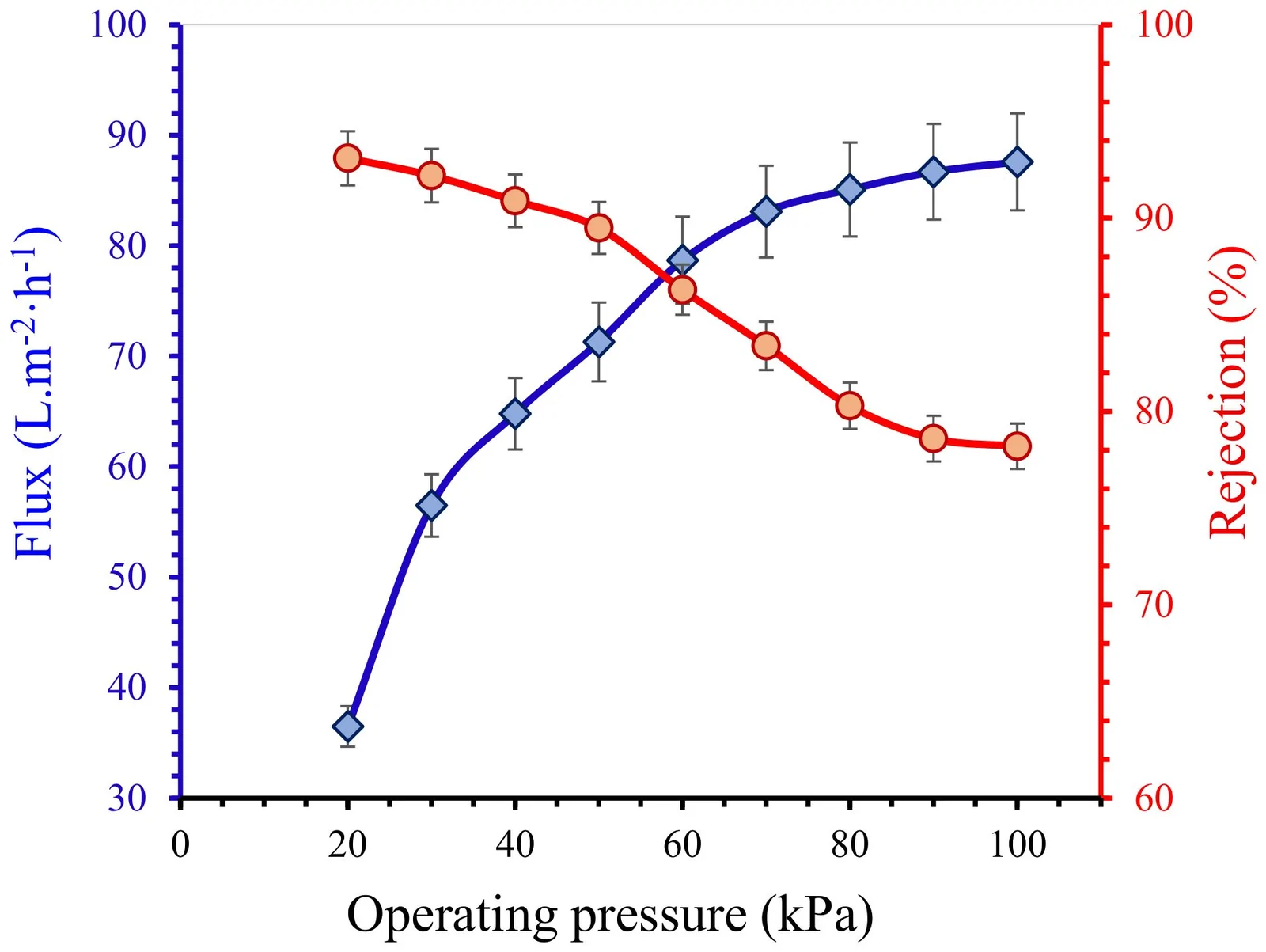
Effect of operating pressure on the oil permeate flux and water rejection of PES-PUMA(4.7) membrane in separation of 10% (*v*/*v*) W/O emulsion [error bars represent the SD of three measurements].

The influence of operating pressure on the oil permeate flux and water rejection efficiency of the PES-PUMA(4.7) membrane is presented in Figure 10. Increasing the operating pressure (from 20 to 100 kPa) led to an increase in oil permeate flux (from 36.5 ± 2.7 to 87.6 ± 3.5 L m^−2^ h^−1^) and a reduction in water rejection (from 93.1 ± 2.1% to 78.2 ± 1.8%). This behavior indicates that higher pressure enhances oil permeation but slightly compromises rejection efficiency.

To quantitatively define a practical operating condition, a multi-response criterion was applied by maximizing oil permeate flux while maintaining water rejection above 85%. Under this constraint, 60 kPa represented the highest investigated pressure that maintained the required rejection level, yielding an oil permeated flux of approximately 78 L m^−2^ h^−1^ and a water rejection of approximately 86.7%. Similarly, increasing the feed flow rate to 0.28 mL s^−1^ provided a substantial flux improvement while maintaining water rejection of approximately 88%. Therefore, 60 kPa and 0.28 mL s^−1^ were identified as representative upper-bound operating conditions for balancing permeation and rejection within the investigated range. This drop in slope at higher pressures points to the limiting effects of concentration polarization and membrane compaction [41]. The decrease in water rejection at higher pressures can be attributed to the increased compressive force on water droplets, which may facilitate droplet deformation and partial penetration into the membrane pores. The membrane maintained high rejection at all tested pressures and showed acceptable separation performance even at low operating pressure [42].

Notably, the PES-PUMA(4.7) membrane maintained high rejection efficiencies across all tested conditions, demonstrating effective separation even at an ultra-low pressure of 20 kPa. This stability up to 60 kPa highlights the membrane’s mechanical robustness for practical applications, although comprehensive assessments of its long-term durability remain a priority for future investigations. The superior performance is associated with the moderate hydrophobicity of the PUMA structure, which effectively hinders the transport of water droplets and surfactants through the membrane matrix.

Statistical evaluation indicated that the uncertainty in the oil permeate flux increased slightly with rising pressure, whereas the uncertainty in water rejection remained nearly constant. Such variations suggest that the oil permeate flux increased slightly as the operating pressure rose from 20 to 100 kPa, whereas the uncertainty in water-rejection efficiency remained nearly constant throughout the investigated pressure range. You et al. (2020) reported that lower flux values in canola oil emulsion treatment are often linked to the presence of smaller oil droplets [43].

To quantitatively describe the effects of operating conditions on membrane performance, quadratic regression models were developed to relate operating pressure and feed flow rate to the two separation responses, namely oil permeate flux and water rejection. The resulting regression equations and related statistical parameters are summarized in Table 2. The negative quadratic coefficients obtained for flux as a function of both operating pressure and feed flow rate indicate a nonlinear relationship, with the incremental increase in flux becoming less pronounced at higher operating conditions. In contrast, water rejection decreased with increasing operating pressure and feed flow rate, indicating a trade-off between membrane permeability and rejection efficiency. These statistical results are consistent with the experimentally observed dependence of membrane performance on the applied operating conditions.

ANOVA was performed to evaluate the statistical significance of the regression models and the relative contributions of the investigated operating parameters. The ANOVA results, including *F*- and *p*-values, are provided in the Supporting Information (Table S5). Operating pressure had a significant effect on both oil permeate flux (*F*-value = 203.95, *p*-value = 3.05 × 10^−6^, *R*^2^ = 0.9855) and water rejection (*F*-value = 111.24, *p*-value = 1.81 × 10^−5^, *R*^2^ = 0.9737). Similarly, feed flow rate significantly affected oil permeate flux (*F*-value = 1664.70, *p*-value = 1.44 × 10^−6^, *R*^2^ = 0.9988) and water rejection (*F*-value = 127.22, *p*-value = 2.40 × 10^−4^, *R*^2^ = 0.9845). For all four models, the regression models were statistically significant (*p*-value < 0.001), with high *R*^2^ values ranging from 0.9737 to 0.9988. The close agreement between *R*^2^ and adjusted *R*^2^ values further supports the adequacy of the regression models in describing the experimental data. These results confirm that both the operating pressure and feed flow rate significantly affect oil permeate flux and water rejection, while the quadratic terms represent the nonlinear response of membrane performance to increasing operating conditions.

**Figure 11:**
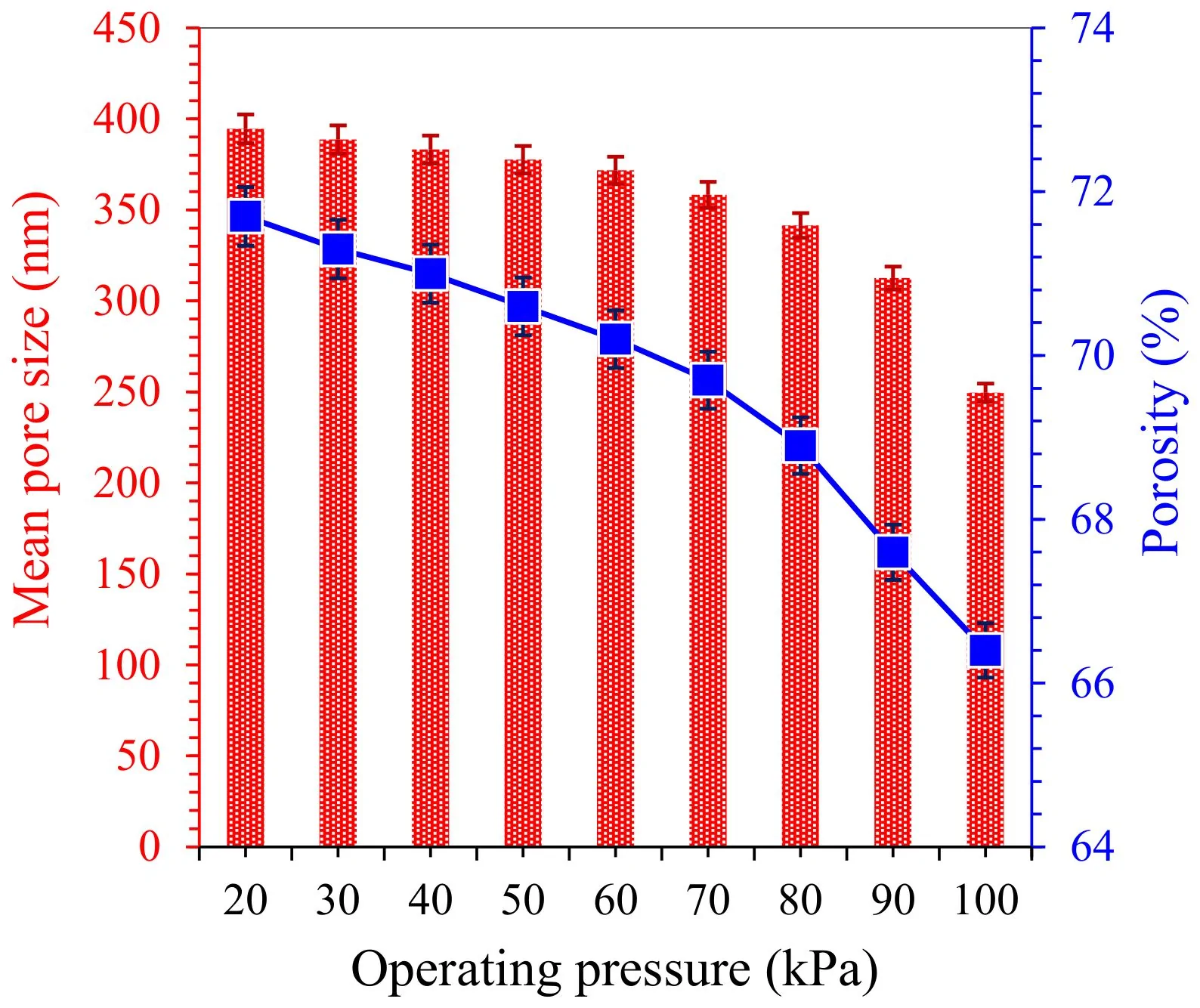
The effect of operating pressure on the mean pore size and porosity of the PES-PUMA(4.7) membrane after each separation cycle of a 10% (*v*/*v*) W/O emulsion [error bars represent the SD of three measurements].

The effect of operating pressure on the porosity of the PES-PUMA(4.7) membrane after each separation of a 10% (*v*/*v*) W/O emulsion is presented in Figure 11. The results indicate that the membrane porosity decreases with increasing operating pressure. This trend is primarily governed by membrane compaction and fouling effects. At an ultra-low pressure (20 kPa), the hydraulic stress exerted on the membrane is relatively low, allowing the membrane to retain most of its pores, leading to a reduction of ~6.5% in mean pore size and ~10.0% in structural porosity. As the operating pressure increases, the greater compressive stress imposed on the membrane matrix induces partial pore deformation, thereby reducing the effective pore volume. At 100 kPa, the reduction in porosity becomes more pronounced owing to the combined influence of membrane compaction, pore blocking, and irreversible fouling effects [44,45].

To evaluate the effect of operating pressure on fouling behavior, the calculated reversible and irreversible flux decline rates for the PES-PUMA(4.7) membrane after ten separation cycles are presented in the Supplementary Materials (Figure S4). The flux decline became more pronounced as operating pressure increased, leading to a higher irreversible and lower reversible components. This result indicates that the higher pressure enhanced foulant deposition and layer compaction on the membrane surface, making a greater portion of the flux loss difficult to recover by physical cleaning. The reversible component was more evident at low to moderate pressures (20–60 kPa), where deposited foulants were more easily removed by hydraulic cleaning. Although the modified membrane structure effectively suppresses excessive foulant accumulation and maintains relatively good pore accessibility, these beneficial characteristics cannot completely prevent pressure-induced membrane compaction, internal pore blockage, and irreversible fouling at high operating pressures.

**Figure 12:**
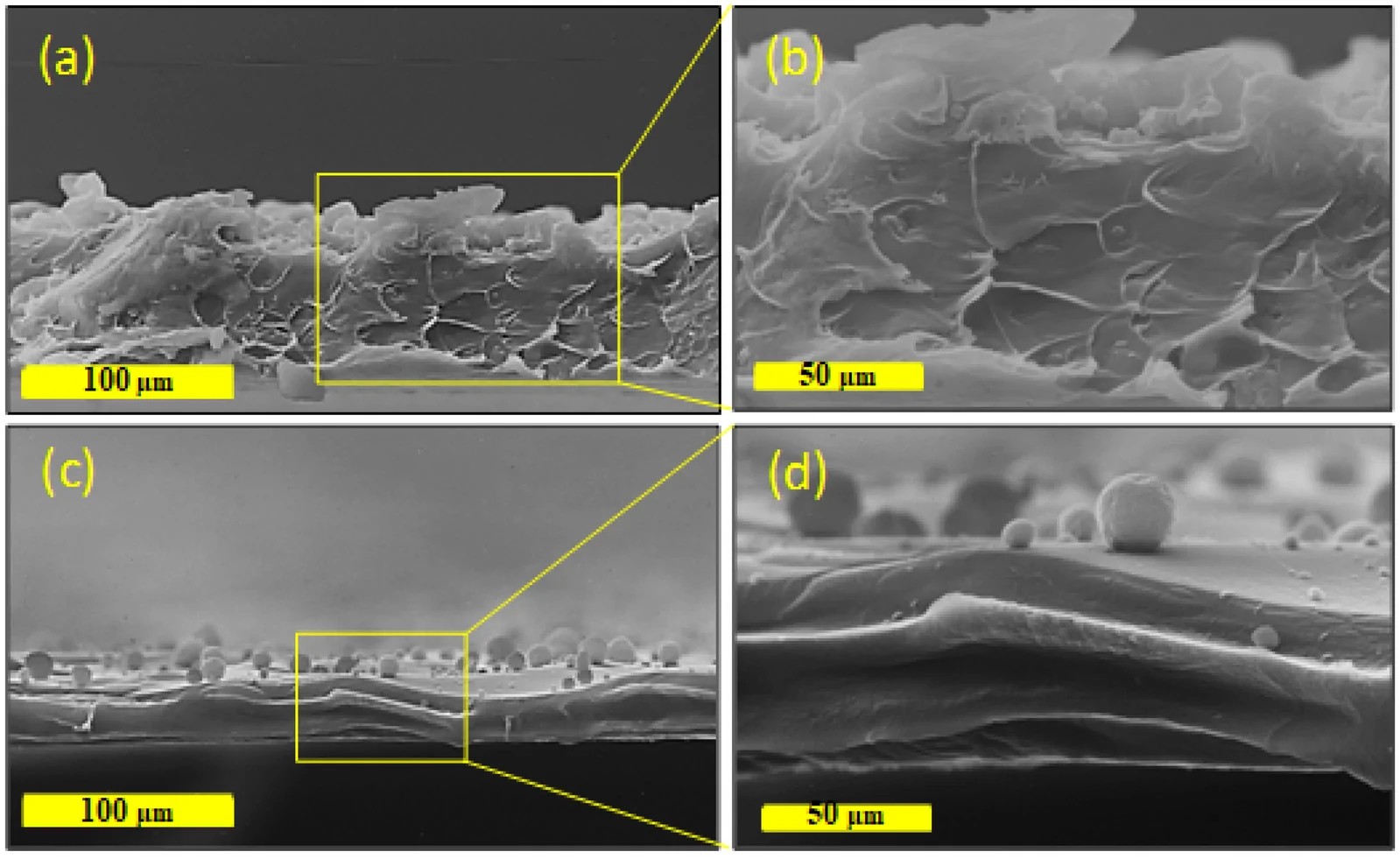
The cross-sectional SEM images of the PES-PUMA(4.7) membrane (**a**,**b**) before, and (**c**,**d**) after oil permeation at an ultra-low pressure of 20 kPa. (**a**,**c**): low-magnification views; and (**b**,**d**) high-magnification views.

The cross-sectional morphologies of the PES-PUMA(4.7) membrane before and after oil permeation at 20 kPa are compared in Figure 12. The membrane exhibits a typical asymmetric structure with interconnected finger-like macrovoids beneath a thin layer (Figure 12a,b). For a direct morphological comparison, the SEM images in Figure 12 were examined at comparable magnification and scale-bar ranges to those reported in our previous study [12]. The differences in the visibility of finger-like macrovoids may therefore largely reflect variations in imaging scale and field of view, although minor morphological variations cannot be completely excluded. After oil permeation within the membrane structure, the cross-sectional morphology remains largely unchanged, indicating that the membrane maintains its structural integrity (Figure 12c,d). However, pores and hollow spaces were found to be filled with foulants (Figure 12c,d).

Additionally, the membrane surface is covered by a compact foulant layer containing numerous retained water droplets and surfactant-stabilized aggregates. The foulants partially block the pores and reduce the effective pore volume, leading to the observed decline in membrane porosity. Notably, no significant deformation or collapse of the finger-like support structure is observed, suggesting that the reduction in porosity primarily results from surface fouling and partial pore blockage rather than permanent structural damage, as supported by the fouling trends in the Supplementary Materials (Figure S3).

#### 3.4.2. Feed Flow Rate Effect

**Figure 13:**
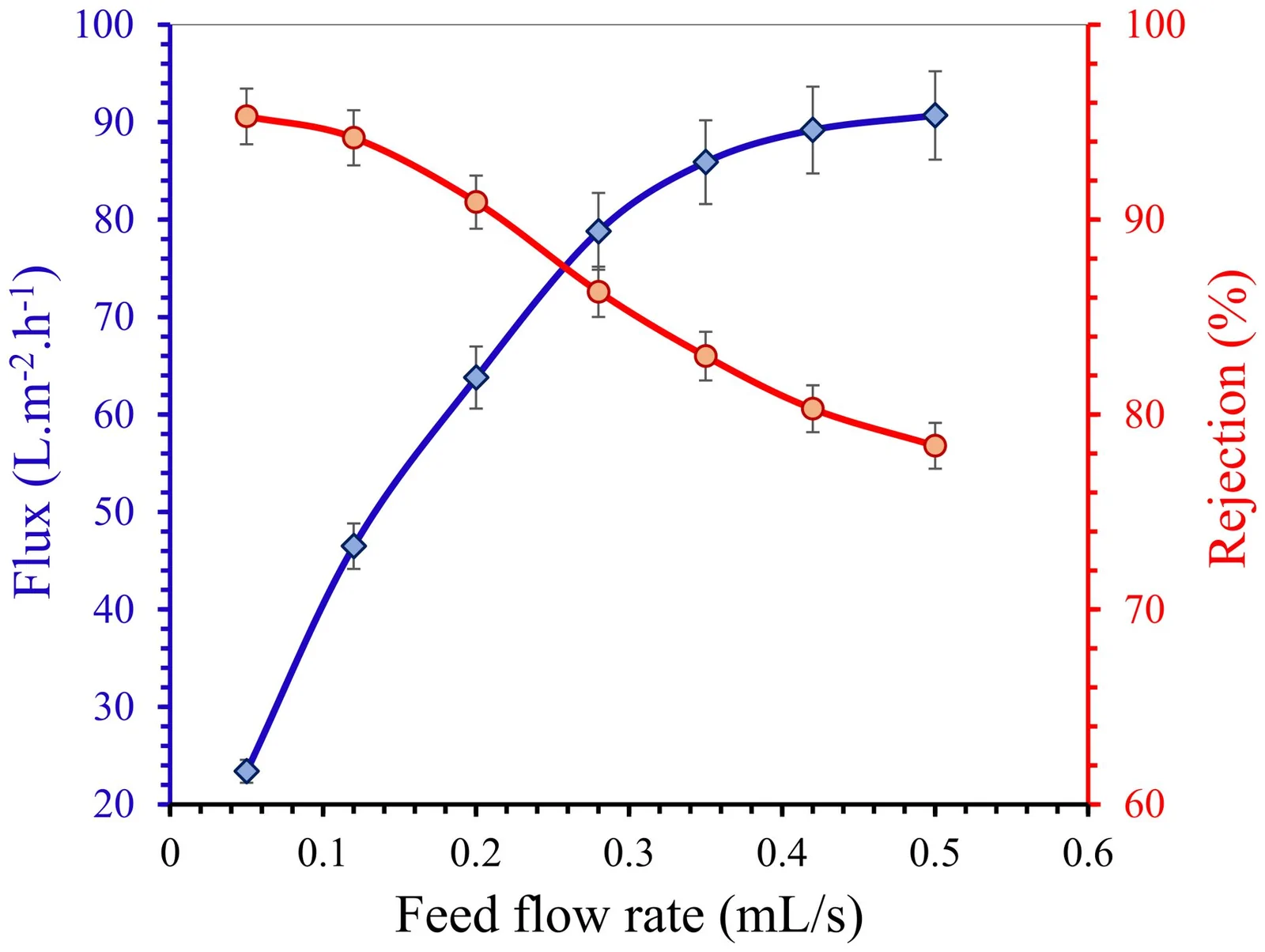
Effect of feed flow rate on the oil permeate flux and water rejection rate of PES-PUMA(4.7) membrane in separation of 10% (*v*/*v*) W/O emulsion [Error bars represent the SD of three measurements].

The oil permeate flux and water rejection efficiency of the PES-PUMA(4.7) membrane as a function of feed flow rate during the separation of a W/O emulsion (10% *v*/*v*) water in paraffin oil are presented in Figure 13. The results show that increasing the feed flow rate significantly enhances the oil permeate flux, particularly at low flow rates, while causing a gradual decrease in water rejection efficiency. This reflects the trade-off between improved mass transfer and reduced separation performance. Increasing the feed flow rate in the range of 0.05–0.12 mL/s led to an increase in oil permeate flux from 23.4 ± 0.7 to 46.5 ± 0.9 L m^−2^ h^−1^. The oil permeate flux increased from 85.9 ± 2.0 to 90.7 ± 2.1 L m^−2^ h^−1^ in the range of 0.35–0.50 mL/s.

The curves show a distinct transition from a steep to more gradual increase in oil permeate flux at a feed flow rate of 0.28 mL/s. These results indicated a shift from a concentration polarization-controlled regime to one dominated by membrane-intrinsic resistance. This behavior is attributed to the reduction in concentration polarization, as the increased crossflow velocity enhances convective mass transfer and shear stress, thereby decreasing the thickness of the polarization layer.

The tendency of the oil permeate flux to approach a plateau with further increases in the feed flow rate indicates that concentration polarization is no longer the primary resistance to mass transport and that additional increases in crossflow provide only limited improvement in oil permeate flux. This behavior is consistent with the literature [46], in which enhanced crossflow hydrodynamics were shown to suppress concentration polarization, reduce external mass transfer resistance, and improve membrane permeation performance.

A decline in water rejection from ~95% to ~78% suggests that stronger hydrodynamic forces may also facilitate droplet deformation and possible partial penetration into surface pores, reducing rejection performance. Therefore, the feed flow rate should be optimized to balance the benefits of reduced concentration polarization and minimized fouling against the potential loss in water rejection. It should also be noted that the membrane’s hydrophilic/hydrophobic structure, the content of the dispersed phase, and the operating conditions can affect the results [47,48].

**Figure 14:**
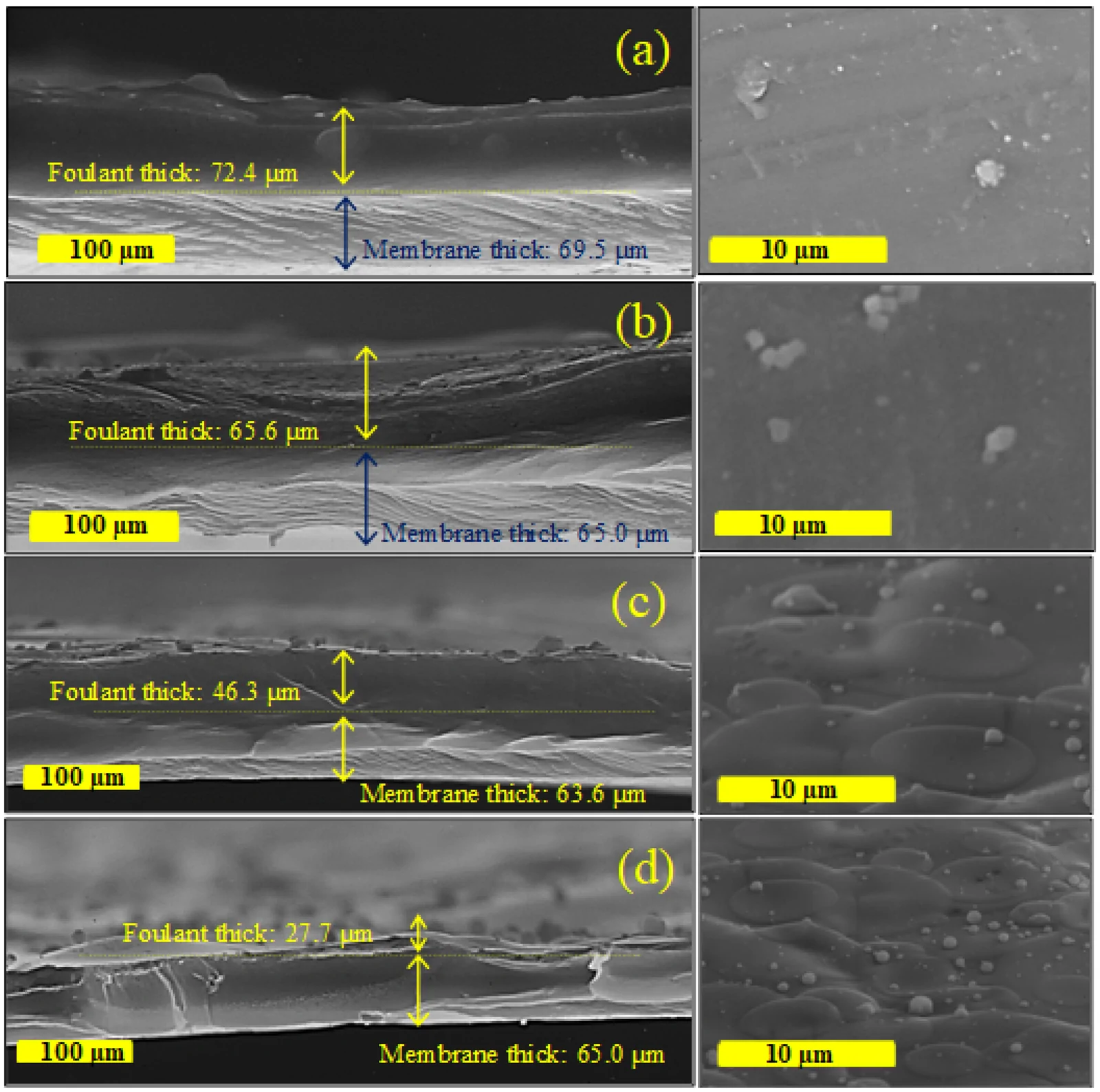
SEM images of PES-PUMA(4.7) membrane after the separation of W/O emulsion (10% *v*/*v*) at feed flow rates of (**a**) 0.05, (**b**) 0.20, (**c**) 0.35, and (**d**) 0.50 mL/s (left and right images represent the cross-sectional, and surface views, respectively).

The effect of feed flow rate on the thickness of the deposited fouling layer was evaluated using cross-sectional and surface SEM images. The SEM images of the PES-PUMA(4.7) membrane after the separation of W/O emulsion (10% *v*/*v*) at feed flow rates of (a) 0.05, (b) 0.20, (c) 0.35, and (d) 0.50 mL/s are shown in Figure 14. The left and right images represent the cross-sectional and surface views, respectively. The cross-sectional SEM images reveal a significant reduction in the thickness of the deposited fouling layer as the feed flow rate increases. The thickness of the foulant layer is 72.4 ± 8.5 μm at the feed flow rate of 0.05 mL/s (Figure 14a). As the feed flow rate increases to 0.20, 0.35, and 0.50 mL/s, the thickness of the foulant layer decreases to 65.6 ± 6.9 μm, 46.3 ± 5.8 μm, and 27.7 ± 4.3 μm, respectively. The observed decrease in thickness could be attributed to the increased hydrodynamic shear stress at higher flow rates [49]. The relatively weak shear force allows the foulants to accumulate more readily on the membrane surface, leading to the formation of a thick and compact cake layer at a low feed flow rate (0.05 mL/s). This thick layer significantly increases the hydraulic resistance [50,51], which leads to a decline in the oil permeate flux. In contrast, increasing the feed flow rate generates higher shear stress at the membrane surface, which effectively removes the deposited foulants from the surface, resulting in a much thinner layer with lower hydraulic resistance. A rougher surface at lower flow rates is due to heavy deposition, whereas higher flow rates help maintain a cleaner membrane surface. In contrast, a thinner fouling layer observed at 0.50 mL/s minimizes the mass-transfer resistance near the membrane surface, leading to improved flux stability and reduced concentration polarization.

#### 3.4.3. Separation Cycle Effects

**Figure 15:**
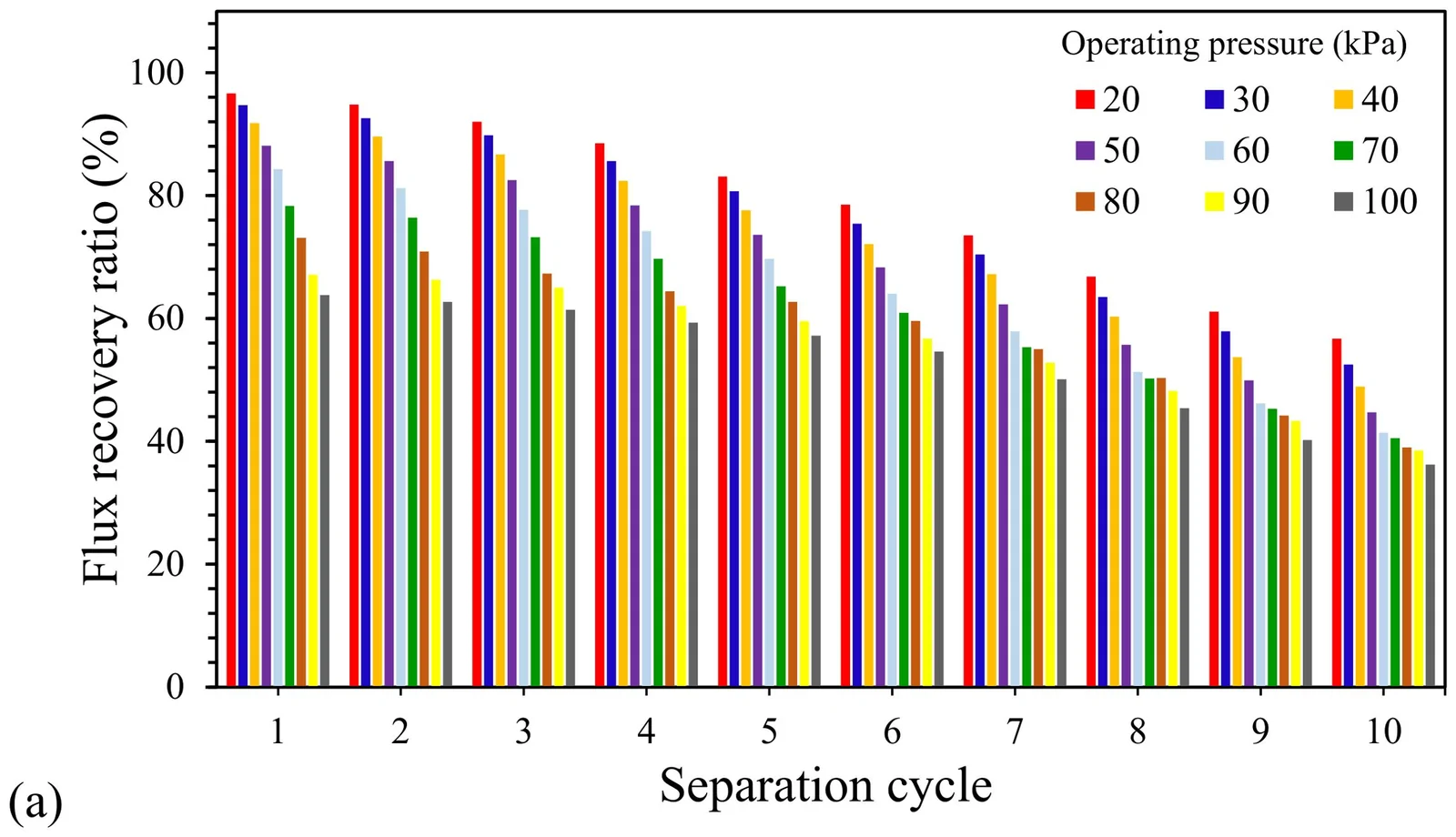

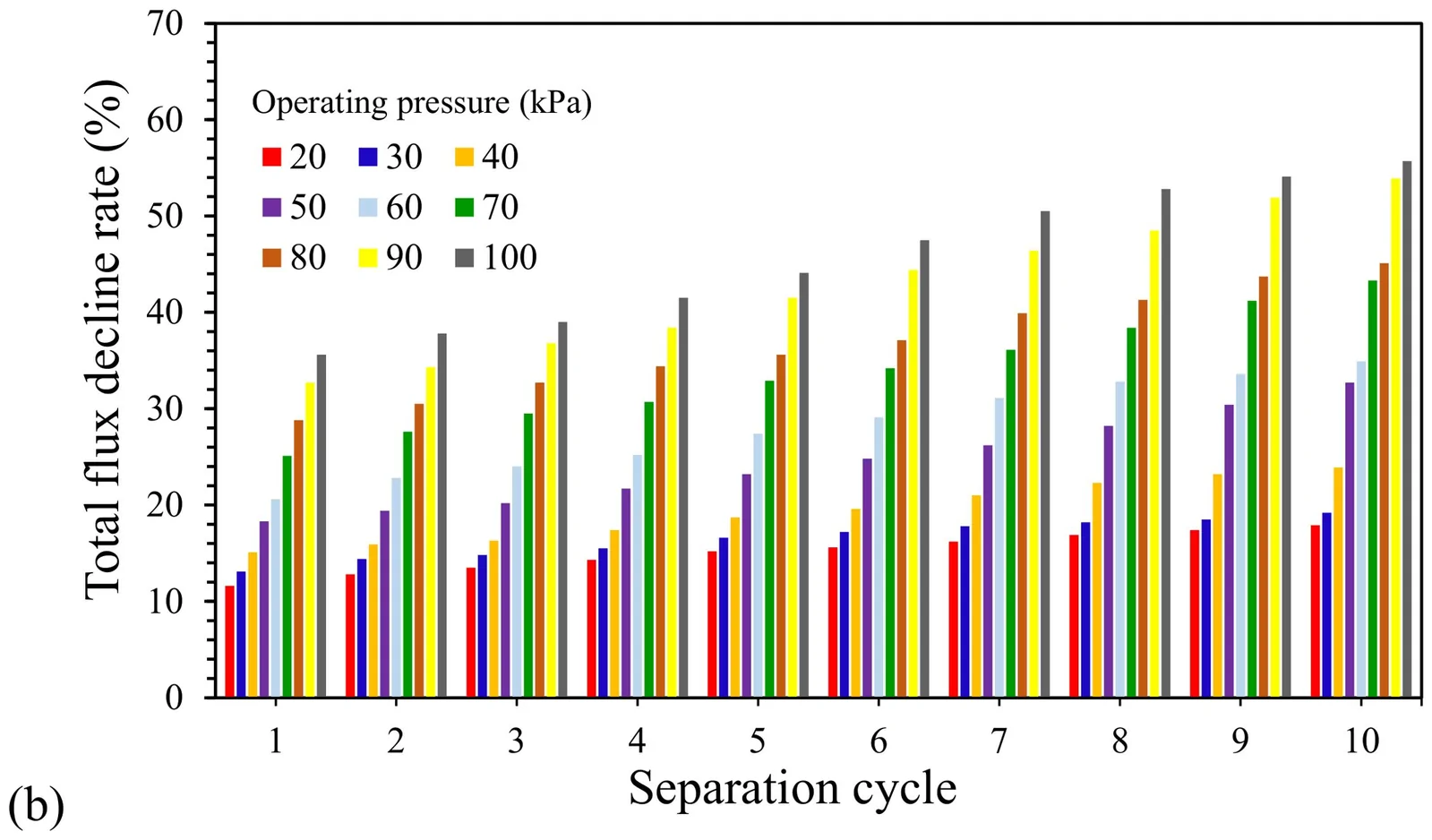
The calculated (**a**) FRR and (**b**) *R*_t_ of PES-PUMA(4.7) membrane values during different separation cycles as a function of operating pressures.

The calculation results of the (a) FRR and (b) *R*_t_ values for the PES-PUMA(4.7) membrane over successive separation cycles at various operating pressures are presented in Figure 15. The separation was performed using a W/O emulsion (10% *v*/*v*) with paraffin oil. PES-PUMA(4.7) was rinsed with *n*-hexane (10 mL) between cycles. The FRR decreased with increasing operating pressure, reaching 36.2% after ten cycles at 100 kPa, indicating severe, irreversible fouling (Figure 15a), whereas the *R*_t_ values increased across the range of 20–100 kPa, demonstrating intensified membrane fouling (Figure 15b).

The relatively weak hydraulic driving force limited foulant transport, resulting in a thin, loosely attached, and largely reversible fouling layer at low operating pressures (20–40 kPa). This resulted in high FRR and low *R*_t_ values, indicating minimal flux loss. As the pressure increased to 40–60 kPa, the higher oil permeate flux promoted concentration polarization, which slightly reduced the FRR.

The observed decline in oil permeate flux at pressures of 60–80 kPa could be attributed to increased cake layer formation and hydraulic resistance. At operating pressures of 80–100 kPa, the fouling layer became increasingly compact due to pressure-induced compression while foulants penetrated deeper into the membrane pores, resulting in the maximum *R*_t_ and lowest FRR values. These findings demonstrate that while higher pressures initially enhance oil permeate flux, they accelerate fouling and intensify flux decline. Another finding is that operating at moderate pressures may provide an optimal balance between oil permeate flux and fouling resistance.

**Figure 16:**
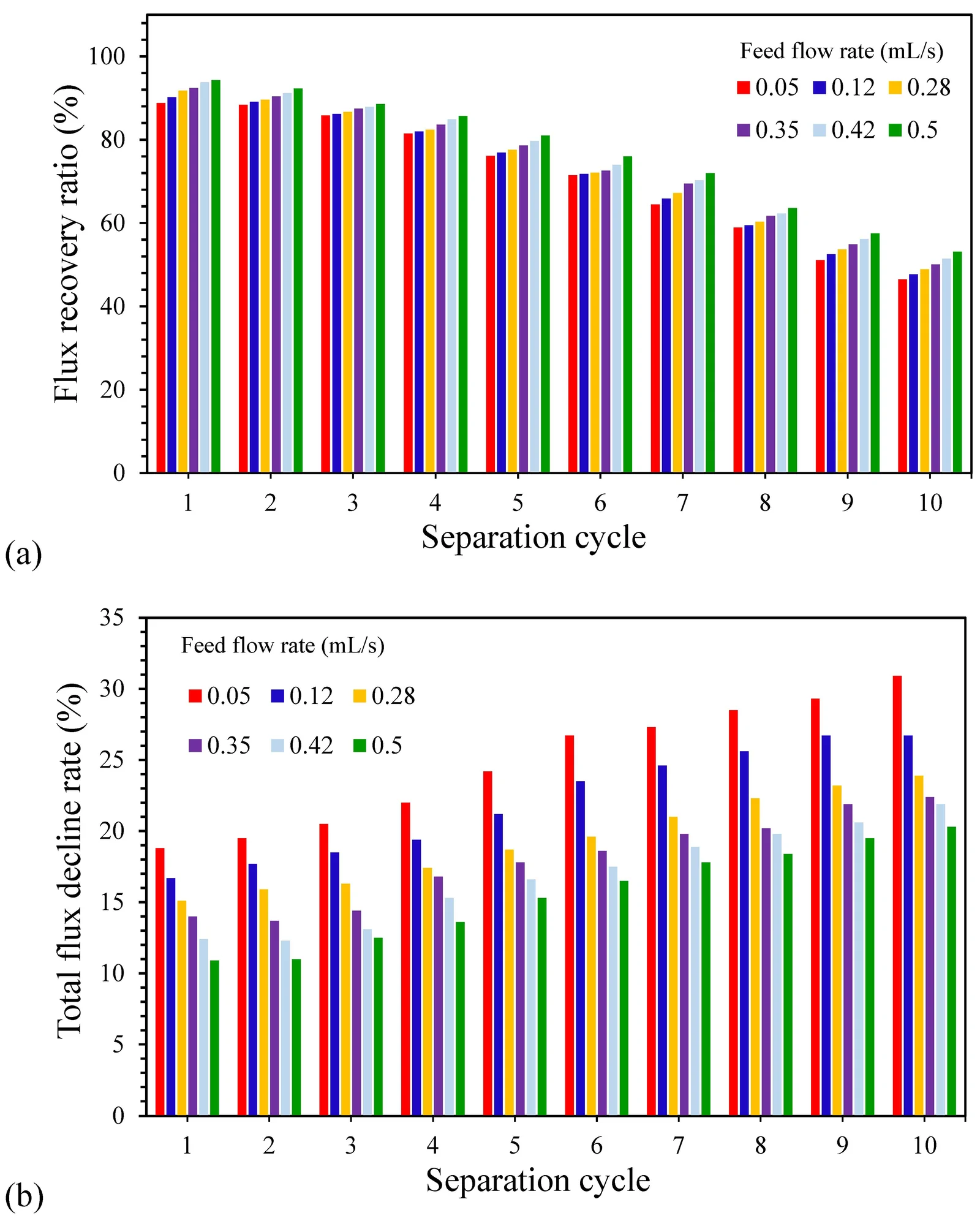
The calculated (**a**) FRR and (**b**) *R*_t_ of PES-PUMA(4.7) membrane values over successive separation cycles at various feed flow rates.

The calculated values of (a) FRR and (b) *R*_t_ of PES-PUMA(4.7) of the PES-PUMA(4.7) membrane over successive separation cycles at various feed flow rates are presented in Figure 16. The FRR value slightly improved with increasing feed flow rate throughout the successive separation cycles (Figure 16a). Lower FRR values were observed at a low flow rate of 0.05 mL/s due to significant foulant accumulation, which reduced hydraulic cleaning. An increase in the feed flow rate led to an enhancement in the shear force at the membrane surface, thereby mitigating foulant deposition and facilitating the removal of loosely attached water foulants. A complementary trend was observed for the *R*_t_ (Figure 16b), which progressively decreased as the feed flow rate increased. The lower *R*_t_ values at higher flow rates indicate that the membrane experienced less overall flux decline during emulsion separation owing to the suppression of concentration polarization and the reduced formation of a compact fouling layer. Although a slight increase in fouling was observed over repeated separation cycles, these results indicate that the PES–PUMA(4.7) membrane maintained stable separation performance and exhibited good resistance to irreversible fouling.

**Figure 17:**
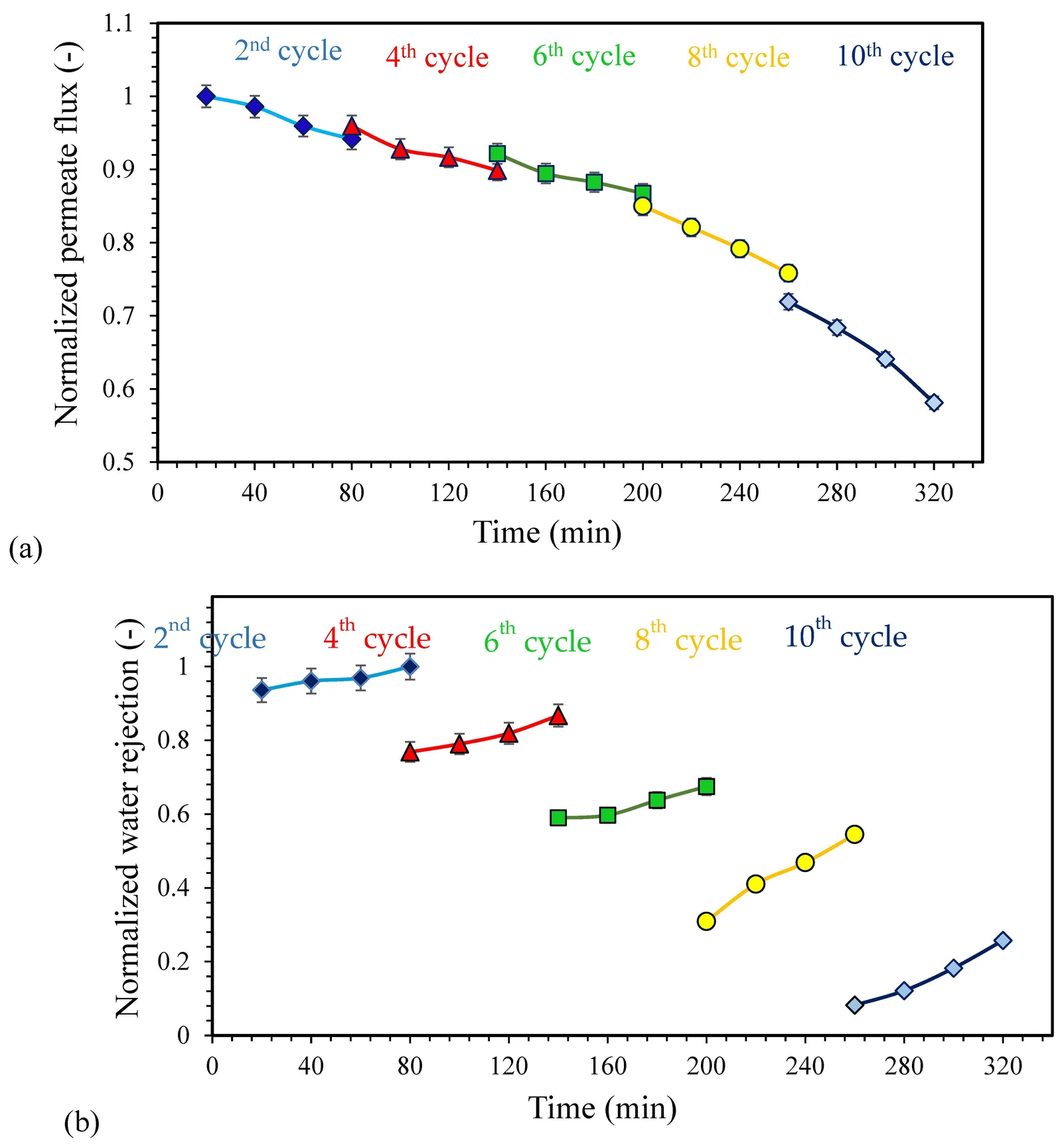
(**a**) Normalized permeate flux and (**b**) normalized water rejection of the PES-PUMA(4.7) membrane during the filtration of W/O emulsion under an operating pressure of 60 kPa.

The normalized oil permeate flux and water rejection of the PES-PUMA(4.7) membrane during the separation of W/O emulsion (10% *v*/*v*) at an operating pressure of 60 kPa are shown in Figure 17. The normalized oil flux of the membrane gradually decreased with increased filtration time, losing 21% and 28% of its initial value after the third and fourth cycles, respectively. The gradual decline in normalized permeate flux during successive filtration cycles indicates progressive fouling and pore constriction. The feed W/O emulsion had a mean droplet size of 754 ± 37 nm, whereas the mean pore diameter of the PES–PUMA(4.7) membrane was approximately 374 nm. Although the broad droplet and pore size distributions preclude direct determination of the fraction of droplets exceeding the pore size, the larger droplets may undergo retention and deposition at the membrane interface, thereby contributing to pore constriction and fouling-layer development during repeated filtration. In addition, the accumulation of residual surfactant and oil at the membrane interface may further promote fouling-layer formation. In contrast, the smaller droplet sizes measured in the permeate (50.6–72.8 nm) represent the fraction that passed through the membrane and should therefore not be interpreted as a primary cause of pore blockage.

The progressive decline in water rejection over successive cycles is consistent with changes in the interfacial fouling layer, droplet deformation, and localized changes in pore accessibility under repeated hydraulic loading. A similar trend was seen for the water rejection results, where a decline in normalized water rejection was seen as the separation cycle increased. This behavior is attributed to the nano-sized water droplets, which may undergo partial deformation or localized coalescence at the membrane interface at moderate pressures, filling the empty pores of the membrane. The increase in oil separation during successive cycles can be attributed to the formation of a foulant layer on the membrane surface.

The cyclic filtration results indicate that the fouling behavior of the PES–PUMA(4.7) membrane is strongly dependent on operating pressure. At low-to-moderate pressures, the deposited foulants remained comparatively more reversible, as reflected by the higher flux recovery and lower total flux decline. In contrast, an increase in the operating pressure led to an increase in the foulant deposition and pore constriction, resulting in progressively lower flux recovery. At 100 kPa, the FRR value decreased to approximately 36.2% after 10 cycles, indicating substantial irreversible fouling under high hydraulic stress. Furthermore, normalized water rejection gradually declined during repeated filtration, suggesting progressive changes in the interfacial fouling layer and pore accessibility. Therefore, although the PES–PUMA membrane exhibited greater fouling reversibility under low-to-moderate pressures, its cyclic stability was not maintained uniformly at elevated pressures. These findings highlight the importance of optimizing hydraulic loading conditions to minimize irreversible fouling and maintain separation performance during repeated operation.

### 3.5. Comparison with the Literature

A comparison of the PES–PUMA(4.7) membrane’s performance with other polymeric membranes, including PES, POPs, polymeric, and ceramic membranes previously analyzed for water-in-oil (W/O) emulsion separation, is presented in Table 3. The membranes were produced through various methods such as phase inversion, electrospinning, and surface modification. Differences in method resulted in changes in membrane morphology and separation performance. Advanced electrospun and nanostructured membranes, including modified and unmodified polyethylene terephthalate (PET) [52], poly(vinylidene fluoride-co-hexafluoropropylene) (PVDF-HFP) [53], covalent organic framework/polyacrylonitrile (COF/PAN) [54], covalent microporous polymer (CMP) nanofibers [55], and polyurethane/titanium carbide (Bio-PU/TiC) [56] exhibit excellent high oil permeate fluxes and water rejection. Nevertheless, multi-step fabrication strategies, the incorporation of hierarchical porous structures, and difficult fabrication methods are obstacles to large-scale production. Additionally, research on PES ultrafiltration and modified Si_3_N_4_ membranes has primarily focused on separation performance, with limited evaluation of long-term fouling behavior, cyclic filtration stability, and flux recovery.

The present study investigates antifouling behavior and filtration resistance as functions of operating pressure over ten consecutive filtration cycles. The results demonstrate that increasing the operating pressure progressively reduces the FRR due to membrane compaction and irreversible pore blockage under severe operating conditions. Furthermore, a PES-PUMA membrane was fabricated through a simple electrospinning-assisted polymer blending strategy, avoiding complex post-modification procedures. To the best of our knowledge, systematic investigations of cyclic fouling dynamics under simultaneous variations in operating pressure and feed flow rate, and their influence on hydraulic stability and filtration resistance, remain scarce in the literature.

### 3.6. Proposed Mechanism for W/O Emulsion Separation by the PES-PUMA Membrane

**Scheme 2:**
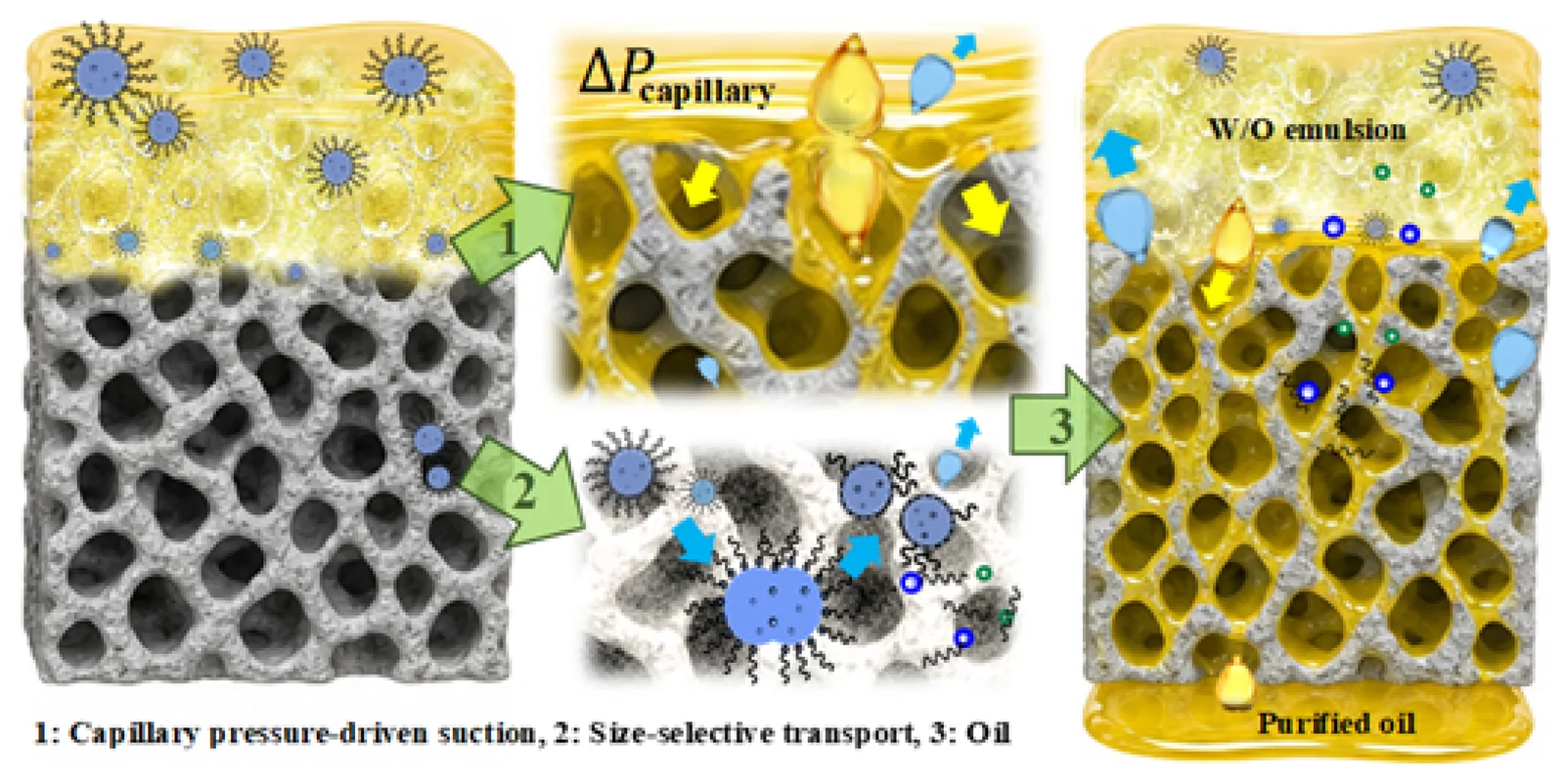
The schematic representation of the mechanism of the W/O emulsion separation by the PES-PUMA membrane.

A schematic representation of the mechanism of W/O emulsion separation by the PES-PUMA(4.7) membrane is shown in Scheme 2. The proposed mechanism consists of (i) capillary pressure-driven oil transport; (ii) size-selective transport of water droplets, with possible droplet deformation or breakup during membrane passage; and (iii) selective oil permeation facilitated by synergistic chemical interactions and capillary trapping of water droplets.

In the first step, the capillary pressure-driven transport of the oil phase is driven by the applied pressure and facilitated by membrane’s porous structure of the PES-PUMA(4.7) membrane. At moderate pressures (40–60 kPa), the applied pressure promotes oil flow through the interconnected pores, while the capillary barrier limits the intrusion of the dispersed phase [61]. The extent of water penetration depends on the pore-throat size, oil–water interfacial tension, and membrane wettability. At higher pressures, water droplets may deform and enter accessible pore regions more readily, which can contribute to the observed decrease in water rejection.

In the second step, the mesoporous structure of PUMA within the PES matrix creates a rough micro- and nanoscale surface with interconnected channels. These features may influence the transport and deformation of water droplets at the membrane interface. The substantially smaller droplet size observed in the permeate (50.6–72.8 nm) compared with the feed emulsion (754 ± 37 nm) is consistent with size-selective transport through the porous membrane. However, droplet deformation or breakup during passage through the pore structure cannot be excluded [62]. Interfacial interactions between Span 80 and the functional groups of PES and PUMA may also influence the stability and transport behavior of the dispersed water droplets. Nevertheless, the present measurements do not include direct dynamic visualization or systematic retentate analysis; therefore, the relative contributions of preferential passage and membrane-induced droplet breakup cannot be conclusively distinguished.

In the third step, the oil permeation process is facilitated by the preferential oil affinity of the PES-PUMA matrix combined with the capillary retention of water droplets within the porous structure. The aromatic segments present in both PES and PUMA provide a chemically compatible environment for oil molecules through London dispersion forces. The integration of PUMA into the PES matrix via hydrogen bonding and π-π stacking interactions may further influence the membrane–oil interactions [63]. Furthermore, the interconnected pore structure promotes oil transport while restricting the passage of larger water droplets. These combined effects contribute to selective oil permeation and water retention, although the extent of long-term pore blockage and its effect on transport should be considered under repeated operating conditions.

## 4. Limitations and Future Perspectives

The results presented herein provide significant insights into the performance of PES–PUMA membranes; however, certain limitations must be acknowledged. This analysis is based solely on a specific range of operating pressures and feed compositions and therefore does not encompass the membrane’s behavior across the full spectrum of industrial hydraulic loads. Additionally, due to practical constraints, this study focused on short-term cyclic filtration performance and did not assess the long-term operational stability required for industrial scaling. Although the chemical integrity of the membrane was confirmed, elucidating the full mechanistic interactions between the polymer matrix and complex foulants is beyond the scope of this work. Future research should aim to expand these boundaries by exploring diverse membrane architectures and conducting pilot-scale studies. Moreover, employing advanced in situ characterization techniques and predictive modeling will be essential to further clarifying fouling mitigation mechanisms.

## 5. Conclusions

The present study builds upon our previous work on PES–PUMA membranes by advancing beyond membrane development and initial material characterization to investigate how hydraulic operating conditions influence separation performance, membrane structural changes, and fouling behavior during cyclic treatment of water-in-oil (W/O) emulsions. The results also indicate that operating pressure and feed flow rate jointly regulate the permeability–water-rejection trade-off, as well as the progression of reversible and irreversible fouling.

The membrane achieved an oil permeate flux of 36.5 ± 2.7 L m^−2^ h^−1^ with a high rejection rate of 93.1 ± 2.1% at 20 kPa. Increasing the pressure to 100 kPa raised the flux to 87.6 ± 3.5 L m^−2^ h^−1^ but reduced water rejection to 78.2 ± 1.8%. This decline was attributed to pressure-induced compaction and pore constriction, as well as droplet deformation and the formation of a relatively compact foulant layer. Similarly, increasing the cross-flow rate from 0.05 to 0.50 mL s^−1^ increased the oil flux from 23.4 ± 0.7 to 90.7 ± 2.1 L m^−2^ h^−1^ while decreasing water rejection from 95.3 ± 2.1% to 78.4 ± 1.2%. Higher cross-flow rates reduced foulant accumulation and limited the development of a persistent foulant layer, thereby enhancing fouling reversibility; however, the associated increase in hydrodynamic shear also intensified the permeability–selectivity trade-off. The quadratic regression models quantitatively describe the nonlinear effects of operating pressure and feed flow rate on oil permeate flux and water rejection. The results demonstrate that hydraulic conditions significantly influence the separation–fouling trade-off and offer a quantitative framework for selecting operating conditions that balance permeability, water rejection, and fouling resistance.

The combined analysis of pore structure, surface morphology, and flux recovery establishes a relationship between pressure, cross-flow, membrane structure, and fouling. This demonstrates that hydraulic conditions actively regulate pore accessibility, foulant deposition, and transport pathways within the membrane. Therefore, the primary contribution of this study is not the re-evaluation of the PES–PUMA material itself but rather the identification of operating-condition-dependent performance and fouling evolution during W/O emulsion separation. The results further indicate that neither increasing pressure nor feed flow rate alone necessarily improves separation performance; instead, an appropriate pressure–feed flow rate operating window is required to balance oil permeability, water rejection, and fouling resistance.

The present study provides a process-level framework for understanding the role of hydraulic operating conditions in hydrophobic PES–PUMA membranes and offers practical guidance for developing stable and efficient membrane processes for oily wastewater treatment and oil–water resource recovery. Further studies involving controlled chemical cleaning and extended filtration–cleaning cycles are required to quantify pore-structure recovery and assess the long-term operational stability of the membrane.

## Figures and Tables

**Table 1:** The results of surface roughness parameters for the PES-PUMA(4.7) membrane after different filtration cycles.

Separation Stage	Roughness Parameters			
	R_p_ (nm) ± SD	R_v_ (nm) ± SD	R_a_ (nm) ± SD	R_q_ (nm) ± SD
Fresh sample	235.6487 ± 11.4482	−129.3511 ± 8.2508	26.1088 ±1.3054	29.8253 ± 1.4871
After 2 cycles	208.007 ± 10.4003	−114.1930 ± 7.0581	22.8075 ±1.2508	26.0071 ± 1.4507
After 4 cycles	153.0336 ± 9.1852	−76.2664 ± 6.5972	12.6752 ±0.8584	16.7542 ± 1.0592
After 6 cycles	139.4854 ± 8.729	−69.5145 ± 5.8904	10.3421 ±0.6250	14.2521 ± 0.9722
After 8 cycles	93.7684 ± 7.9872	−40.6316 ± 4.4806	8.8647 ± 0.4485	10.2973 ± 0.7508
After 10 cycles	88.5129 ± 6.9708	−35.4871 ± 3.5870	6.4810 ± 0.3015	8.6544 ± 0.4681

**Table 2:** Proposed regression models and ANOVA statistical results.

Response	Hydraulic Variable	Quadratic Regression Model	*R* ^2^	*F*-Value	*p*-Value
Oil permeate flux, J (L m^−2^ h^−1^)	Operating pressure (kPa)	J = 8.284 + 1.775 *P* − 0.0099 *P*^2^	0.9855	203.954	3.05 × 10^−6^
Water rejection, R (%)	Operating pressure (kPa)	R = 97.241 − 0.157 *P* − 0.00046 *P*^2^	0.9737	111.237	1.81 × 10^−5^
Oil permeate flux, J (L m^−2^ h^−1^)	Feed flow rate (mL s^−1^)	J = 6.841 + 367.683 *Q* − 402.7130 *Q*^2^	0.9988	1664.696	1.44 × 10^−6^
Water rejection, R (%)	Feed flow rate (mL s^−1^)	R = 98.336 − 42.540 *Q* + 2.5170 *Q*^2^	0.9845	127.222	2.40 × 10^−4^

**Note:** *P* is the operating pressure (kPa), and *Q* is the feed flow rate (mL s^−1^). The quadratic terms describe the nonlinear dependence of membrane performance on these hydraulic parameters.

**Table 3:** Comparison of the performance of PES membranes, POPs, polymers and inorganic materials for the separation of W/O emulsions.

Entry	Membrane/ Material	Fabrication Method/ Emulsion Type	Operational Parameters	Flux (L m^−2^ h^−1^)	Rejection (%)	FRR (-)	Antifouling; Chemical Stability	Ref
1	PES ultrafiltration membrane (10–300 kDa)	Phase inversion/20% mineral oil, 2% Span, 78% DI water	0.05–0.30 MPa, 0.1–2.0 L min^−1^	NR	NR	NR	NR	[57]
2	Ceramic membrane modified with α-Si_3_N_4_ nanowires/organosilane NPs	Surface modification/ 1 vol% DI water, Span 80, Kerosene	0.02, 0.05, 0.1, 0.15 and 0.20 MPa, 0.1–2.0 L min^−1^	NR	>95	NR	Good; NR	[58]
3	PET fibrous membrane	Electrospinning followed by surface modification/5% water, 1% Span 80, 94% CCl_4_	0.0008 MPa 0.0012 MPa	4395 >1100 (gravity-driven)	>92 99.0–99.9 (gravity-driven)	NR	>95% after 40 cycles; Good	[59]
4	Unmodified recycled PET nanofibrous membrane	Electrospinning/ 95 vol% hexadecane, 5 vol% DI water, Span 80 (1 wt%)	Vacuum-assisted filtration	>6500	>98 fresh >95 after 3 cycles	NR	3 cycles; High stability in acidic/alkaline media	[52]
5	Bio-PU/TiC	Electrospinning/10 vol% water, Span 80 and 90 vol% hexane	Pressure-assisted/gravity-assisted	2464.79	99.65	NR	NR; Good	[56]
6	PVDF/HFP	Electrospinning/water: 1 wt%, 99% *n*-hexane and diesel oil	Vacuum: −0.08 MPa	2436–25,000	99.63–99.95	NR	Good; NR	[53]
7	COF-haTab/PAN	Electrospinning/Span 80, 1–10 wt% water, 90 vol% *n*-hexane	Gravity-driven filtration	4229	>99.9	NR	Excellent; Good	[54]
8	CMPs	Electrospinning/Span 80, 1 vol% water, 99 vol% Dichloromethane	Vacuum-assisted filtration	865.8	99.9	NR	NR; Good resistance to dichloromethane	[55]
9	PES modified with fluorinated SiO_2_ NPs	Phase inversion/10 vol% water, 90 vol% *n*-hexane	Dead-end vacuum	1020.4	97.4	NR	5 cycles	[60]
10	PES–PUMA(4.7)	Electrospinning/water: 10 vol%, 90 vol% oil	0.02–0.1 MPa, 0.05–0.5 mL/s	36.5–87.6 23.4–90.7	78.2–93.1 78.4–95.3	63.8–96.6	10 cycles; Excellent (Acid/Alkali)	This work

NR: Not reported.

## Data Availability

The original contributions presented in this study are included in the article/Supplementary Material. Further inquiries can be directed to the corresponding author.

## Supplementary Materials

The following supporting information can be downloaded at: https://www.mdpi.com/article/10.3390/membranes16100350/s1. The supplementary material includes the physical characteristics of paraffin oil, description of characterization procedure of membrane and W/O emulsion, cross-sectional SEM image of PES-based membranes, and the results of the reversible and irreversible components of PES-PUMA(4.7) membrane.
