# Supplementary material for: Pressure and Cross-Flow Dependent Interfacial Fouling of Mesoporous PES-PUMA Membranes During Water-in-Oil Emulsion Separation
Source: Membranes (Basel). 2026 Oct 21;16(10):350. doi: 10.3390/membranes16100350 (PMC13707103; doi:10.3390/membranes16100350)
Supplement: Supplementary file 1 [file membranes-16-00350-s001.zip › membranes-4601079-supplementary.pdf]

Supplementary Materials

Pressure and Cross-Flow Dependent Interfacial Fouling of Mesoporous PES-PUMA Membranes during Water-in-Oil Emulsion Separation

Alireza Bahramian[1]* and Sedigheh Alavinia[2]

[1] *Chemical Engineering Department, Hamedan University of Technology, Hamedan 65155, Iran*

[2] *Department of Organic Chemistry, Faculty of Chemistry and Petroleum Sciences, Bu-Ali Sina University, Hamedan, Iran.*

* *Corresponding author:* bahramian@hut.ac.ir

1. A summary list of chemicals and materials used in this study

Table S1. List of chemicals and materials employed in membrane fabrication and emulsion preparation.

Material / Reagent	Purity (%)	Supplier
1,4-Phenylene diisocyanate	99	Merck, Germany
1,3,5-Triazine-2,4,6-triamine (MA)	99	Merck, Germany
Dimethylformamide (DMF)	98	Merck, Germany
Triethylamine	99	Merck, Germany
Hydrofluoric acid (HF)	40	Merck, Germany
Polyethersulfone (PES, Radel® A300)	99	Amoco Chemical
Ethanol	98	Sigma-Aldrich, USA
Ammonia solution	29	Sigma-Aldrich, USA
Tetraethyl orthosilicate (TEOS)	98	Merck, Germany
Span 80 (Sorbitan monooleate)	>99	Merck, Germany
Sodium carbonate	>99	Merck, Germany
Sodium chloride	>97	Merck, Germany
Deionized water (Milli-Q)	18 MΩ.cm	Merck Millipore
Paraffin oil	99	Sarvestandarou Co., Iran

2. Fabrication of SiO_2/PUMA nanocomposite, membrane and characterization procedure description

2.1. Fabrication of SiO_2/PUMA nanocomposite

The presence of unique porous PUMA structures in the absence of SiO_2 NPs confirms the successful etching of the silica template. The SiO_2/PUMA nanocomposite was subsequently prepared *via* sol–gel polymerization following a previously reported procedure. Briefly, a mixture of MA (0.8 mmol) and 1,4-phenylene diisocyanate (1 mmol) was dissolved in 30 mL of DMF. SiO_2 NPs (0.007 g) were then added and dispersed uniformly. Subsequently, triethylamine (0.1 mL) was slowly added dropwise to the reaction mixture, followed by continuous stirring at 300 rpm and stirred at 100 °C for 6 h. The product was then washed alternately with a 1:1 ethanol–water mixture (50 mL), and dried under reduced pressure at room temperature.

2.2. Electrospinning parameters

The optimization of electrospinning parameters, including applied voltage, needle-to-collector distance, flow rate, and PUMA concentration is presented in Table S1. Based on the optimization results, the best nanofibers were obtained at a distance of 20 cm from the nozzle at an injection rate of 5 mL per hour, and a voltage of 20 kV with 4.7% PUMA. The electrospinning process was performed at room temperature for 50 min. The applied voltage was 20 kV, with a tip-to-collector distance of 20 cm. Therefore, the electric field strength calculated from (E = V/d) is approximately 1 kV cm^{-1}, rather than 20 kV cm^{-1}.

Table S2. Electrospinning parameters in the membrane fabrication.

Applied voltage	20 kV
Electric field strength	1 kV cm^{-1}
Flow rate	5 $mL.h^{-1}$
Needle orientation	Horizontal
Needle–collector distance	20 cm
Collector type	Rotating plate
Electrospinning time	50 min
Temperature	298 K
Relative humidity	35% RH

2.3. Determination of oil permeability, water removal efficiency, and fouling parameters

The oil permeation flux (J_o, L.m^{-2}.h^{-1}) was calculated by the following equation:

$$J_o = \frac{V}{A\times \Delta t} \quad \text{(S.1)}$$

where V is the volume of permeated oil (L), A represents the effective membrane area (m^2), and Δt is the permeation time (h).

The separation efficiency (E) of the prepared membranes was determined according to the following equation:

$$E = \left(1 - \frac{C_1}{C_0}\right) \times 100\% \quad \text{(S.2)}$$

where C_0 and C_1 are the water concentration before and after the separation process, respectively.

The flux recovery ratio (FRR) was estimated by analyzing the time-dependent flux variations during the filtration process to quantify the antifouling characteristics of the prepared membranes, using the following equation:

$$FRR = \left(\frac{J_{o,n}}{J_{o,i}}\right) \times 100\% \quad \text{(S.3)}$$

where $J_{o,i}$ and $J_{o,n}$ represent the initial pure oil flux and pure oil flux after washing at the end of n cycle (n = 1-4), respectively.

The fouling resistance parameters, including the total flux decline rate (R_t), reversible flux decline ratio (R_r) and irreversible flux decline ratio (R_{ir}) were used to evaluate the antifouling performance of the membranes. These parameters were calculated using the following equations:

$$R_t = \left(\frac{J_{o,i}-J_{e,n}}{J_{o,i}}\right) \times 100\% \quad \text{(S.4)}$$

$$R_r = \left(\frac{J_{o,n}-J_{e,n}}{J_{o,i}}\right) \times 100\% \quad \text{(S.5)}$$

$$R_{ir} = \left(\frac{J_{o,i}-J_{o,n}}{J_{o,i}}\right) \times 100\% = R_t\text{-}R_r \quad \text{(S.6)}$$

where $J_{e,n}$ represent the steady-state permeate flux of emulsion during n cycle.

2.4 Membrane Characterization

2.4.1. Morphology and microstructure

The surface and cross-sectional morphologies of the PES-based and PES–PUMA membranes were characterized using scanning electron microscopy (SEM; JSM JEOL 840A) at an accelerating voltage of 30 kV. The size and morphology of the SiO_2 NPs and dispersed PUMA structure were analyzed using field-emission scanning electron microscopy (FE-SEM; FEI Quanta 300 FEG).

The surface morphology, thickness, and roughness of the membranes were characterized using atomic force microscopy (AFM; Bruker). The images depict the topography and height of the electrospun nanofibers obtained within a 10 µm × 10 µm frame as both 2D and 3D AFM scans. The morphology of the film surface was analyzed using the Scanning Probe Image Processor (SPIP) software package, which calculated the average values of several roughness parameters over the measured area. A number of standard surface roughness parameters used in this study are introduced in the following:

Average roughness (R_a)

$$R_a = \frac{1}{MN}\sum_{j=0}^{M-1}\sum_{i=0}^{N-1}\eta(x_i, y_j) \quad \text{(S.7)}$$

Root mean square roughness (R_q)

$$R_q = \sqrt{\frac{1}{MN}\sum_{j=0}^{M-1}\sum_{i=0}^{N-1}\eta^2(x_i, y_j)} \quad \text{(S.8)}$$

M: is a number of points of per profile (scan line)

N: is the number of profiles

η: amplitude at (x_i, y_j)point

Valley value (R_v)

$$R_v = |\min \eta(x_i, y_j)| \quad \text{(S.9)}$$

$\eta(x_i, y_j)$is the surface height (amplitude) at the point (x_i, y_j).

min represents the minimum value of the surface height (the deepest valley on the surface).

Peak value (R_p)

$$R_p = |\max \eta(x_i, y_j)| \quad \text{(S.10)}$$

$\eta(x_i, y_j)$is the surface height (amplitude) at the point (x_i, y_j).

max represents the maximum value of the surface height (the highest peak on the surface).

2.4.2. Chemical characterization

Fourier-transform infrared (FT-IR) spectroscopy (PerkinElmer Spectrum 65) was used to analyze the chemical structure and surface functional groups of the membranes. In addition, FT-IR spectra of the PES–PUMA membranes were collected after pure oil permeation and after exposure to W/O emulsions to detect possible spectral changes related to oil permeation and membrane–foulants interactions.

2.4.3. Actual pore size and porosity measurements

The mean pore size and pore size distribution were determined using the gas-flow/liquid displacement method (Porometer, Porolux 1000). Before measurements, the membrane samples, with a diameter of 30 mm, were fully wetted with Porefil liquid (IB-FT GmbH, Germany, density 1.87 g/mL) for 15 min. The experiments were performed using the "wet up-dry up" method at 25 °C, in which the liquid dewetting was followed by measuring the gas flow as a function of pressure. The mean pore size of the prepared membranes was estimated using the porometer software.

The porosity of the prepared membranes was determined by the gravimetric method using anhydrous ethanol as the wetting liquid. The dry membrane weight (W_d) was first measured, after which the membrane was immersed in anhydrous ethanol for 1 h. The wet membrane weight (W_w) was then measured after gently removing excess liquid from the surface. The membrane porosity, ε, was calculated using the following:

$$\varepsilon(\%) = \frac{W_w - W_d}{\rho_{\mathrm{EtOH}} \times V_d} \times 100 \qquad \text{(S.11)}$$

where ρ_{EtOH} is the density of anhydrous ethanol $(0.789\ \mathrm{g\,cm^{-3}})$, and V_d is the bulk volume of the dry membrane.

2.4.4. Crystallinity and thermal stability

The crystalline structure of the membranes was studied by X-ray diffraction (XRD) analysis (Philips PW 1710 diffractometer with Cu Kα radiation (λ = 1.54056 Å). The corresponding curves were obtained over a 2θ range of 20° to 80°, with a step size of 0.04°.

Thermal stability was assessed by thermogravimetric analysis and derivative thermogravimetry (TGA/DTG) analyzer (Perkin-Elmer Instrument, Pyris 1). The heating behavior of prepared samples were determined by a differential scanning calorimetry (DSC) analyzer (Netzsch-Gerä tebau GmbH, 200F3 Maia ® model). DSC analysis was carried out under an inert atmosphere in the temperature range of 20–250 °C at a heating rate of 10 °C $\mathrm{min^{-1}}$. A preheating step was applied before the DSC measurement to remove thermal background effects and minimize the influence of prior thermal history.

2.5. Emulsion characterization and wetting measurements

The droplet size distribution of the W/O emulsions was measured by dynamic light scattering (DLS; HORIBA SZ-100-S2). The initial oil contact angle (OCA), water contact angle (WCA), and early-stage wetting behavior were determined using a goniometer (DSA-100, Krüss). To visualize droplet penetration and dynamic wetting behavior, 5 µL droplets of paraffin oil and deionized water were deposited on the membrane surface, and their infiltration process was recorded using a high-magnification digital microscope (1000× USB HD). These observations enabled direct monitoring of oil infiltration pathways and the temporal evolution of water repellency. Residual water content in the permeate was measured utilizing a Karl Fischer titrator (Eco KF Titrator, Metrohm, Switzerland) equipped with an integrated magnetic stirrer. All measurements were conducted in accordance with the ASTM D6304 standard (2020/2021 revision).

All measurements were carried out in triplicate, and the results are presented as mean ± standard deviation (SD). The OCA and WCA measurements showed excellent reproducibility, with a standard deviation below 0.2°, whereas the values extracted from microscopic image sequences exhibited slightly larger deviations (1–3°), which is typical for image-based wetting-dynamics analysis.

3. The physical properties of paraffin oil and W/O emulsions

Table S3. The main physical properties of paraffin oil.

Relative density<br>(g/cm^3, 20 °C/water at 20 °C)	0.914-0.920
Refractive index (40 °C)	1.465-1.467
Viscosity (kinematic viscosity at 20 °C, mm^2/sec)	78.2
Specific heat (J/g at 20 °C)	1.910-1.976

Table S4. The dynamic viscosity of W/O emulsion samples at 20 °C

Emulsion sample	Dynamic viscosity (cP)
W/O (10 vol% H_2O)	32
W/O (15 vol% H_2O)	75
W/O (20 vol% H_2O)	156
W/O (25 vol% H_2O)	363
W/O (30 vol% H_2O)	667

4. Antifouling property and cyclic stability analysis

The antifouling characteristics of the membranes were estimated by the flux recovery ratio (*FRR, %*), calculated from time-dependent flux variations during the cyclic filtration process, as shown in the following equation:

$$FRR = \left(\frac{J_{o,n}}{J_{o,i}}\right) \times 100\% \qquad \text{(S.3)}$$

where $J_{o,i}$ denotes the initial pure-oil flux, and $J_{o,n}$ represents the pure-oil flux measured after membrane washing at the end of nth cycle (n = 1-10)

The fouling resistance parameter of membrane during different separation cycles was determined by measuring the oil permeate flux of clean, fouled, and cleaned membranes. This approach allows the quantification of the total flux decline ratio (R_t), which was calculated using the following equation:

$$R_t = \left(\frac{J_{o,i}-J_{e,n}}{J_{o,i}}\right) \times 100\% \qquad \text{(S.4)}$$

The reversible flux decline ratio (R_r) and irreversible flux decline ratio (R_{ir}) were used to evaluate the fouling effect on the membrane performance. These parameters were calculated using the following equations:

$$R_r = \left(\frac{J_{o,n}-J_{e,n}}{J_{o,i}}\right) \times 100\% \qquad \text{(S.5)}$$

$$R_{ir} = \left(\frac{J_{o,i}-J_{o,n}}{J_{o,i}}\right) \times 100\% = R_t\text{-}R_r \qquad \text{(S.6)}$$

where $J_{e,n}$ represents the steady-state oil permeate flux of the emulsion during the nth separation cycle.

5- Surface morphology of SiO_2 NPs and PES-based membranes

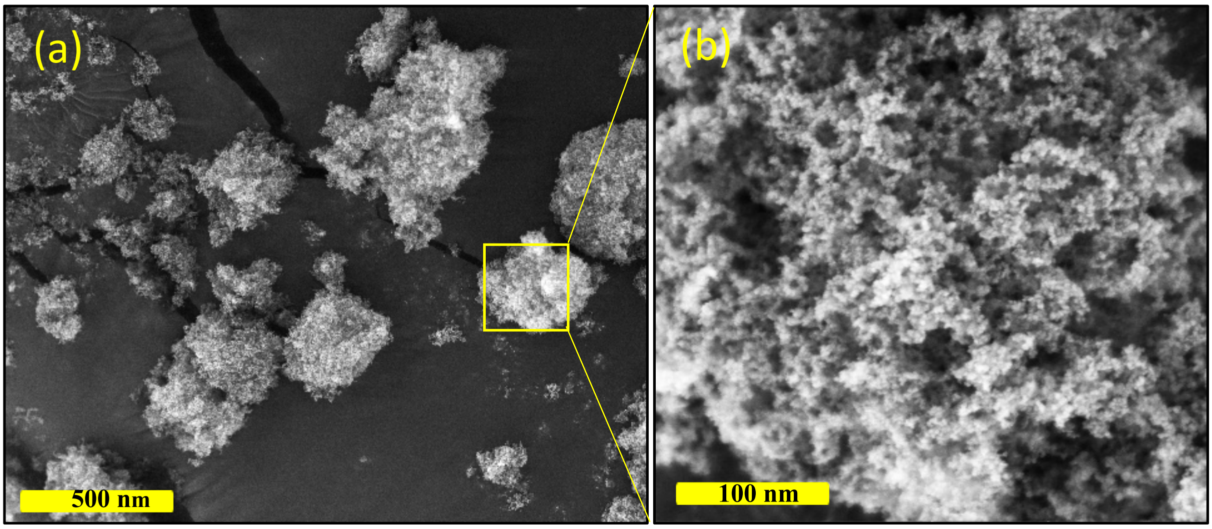

Figure S1. The surface morphology of SiO_2 NPs (a: low-magnification, b: high-magnification images)

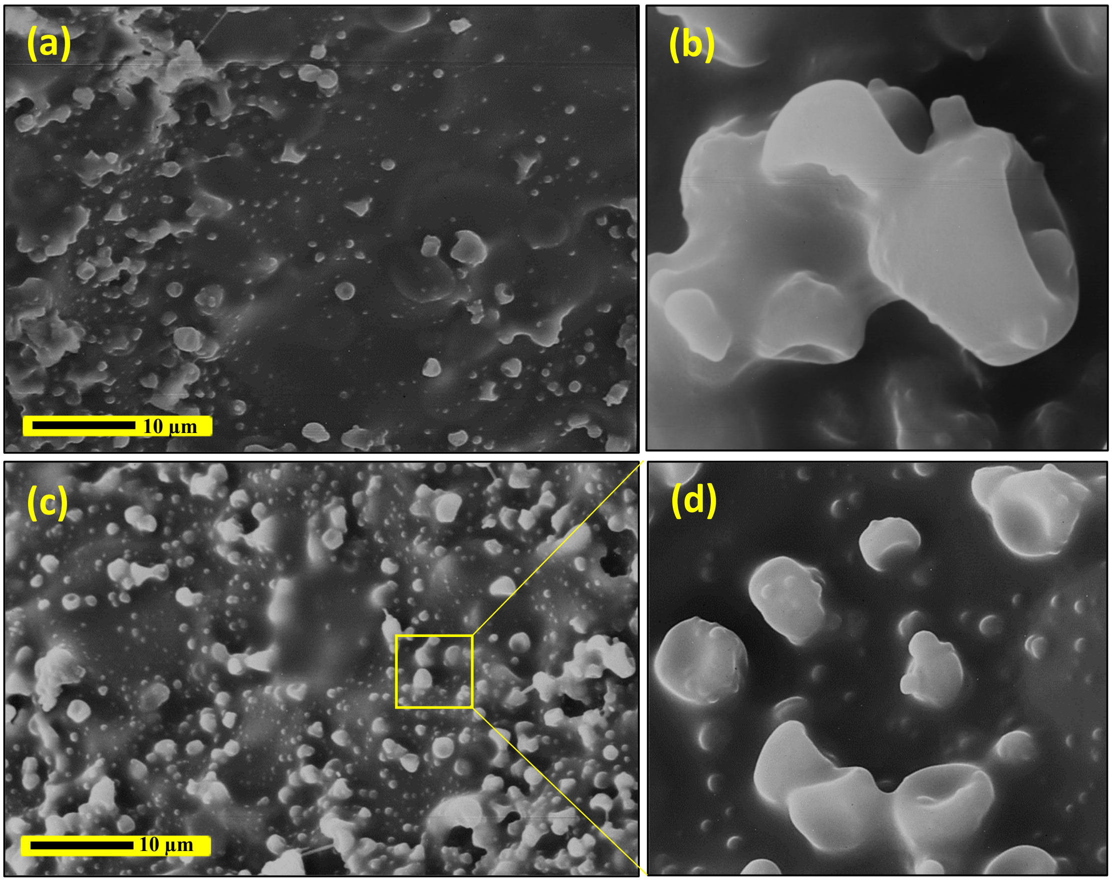

Figure S2. The surface morphology of (a and b) PES-PUMA (1.2) and PES-PUMA (2.5) membranes with (a and c) low- and (b and d) high-magnification images.

6- Cross-sectional view of PES-based membranes

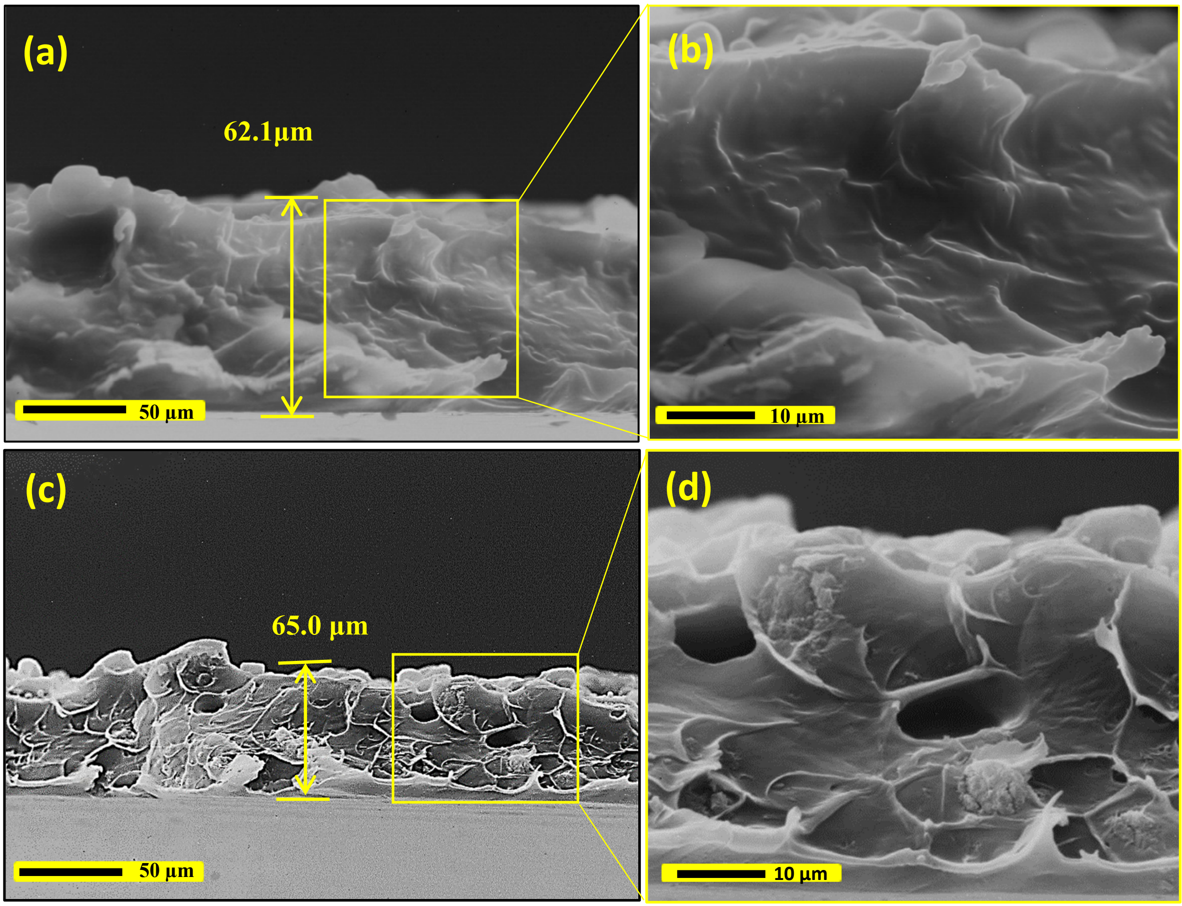

Figure S3. The cross-sectional SEM image of PES-PUMA(1.2) and PES-PUMA(2.5) membranes with (a and c) low- and (b and d) high-magnification images.

7. ANOVA results for the effects of operating pressure and feed flow rate on membrane performance.

Table S5. ANOVA results for the effects of operating pressure and feed flow rate on the membrane performance.

**Factor**	**Response**	**Model**	R^2	***F*-value**	***p*-value**
Operating pressure	Oil permeate flux	Quadratic	0.9855	203.95	3.05×10^{-6}
Operating pressure	Water rejection	Quadratic	0.9737	111.24	1.81×10^{-5}
Feed flow rate	Oil permeate flux	Quadratic	0.9988	1664.70	1.44×10^{-6}
Feed flow rate	Water rejection	Quadratic	0.9845	127.22	2.40×10^{-4}

8. The reversible and irreversible components of PES-PUMA(4.7) membrane

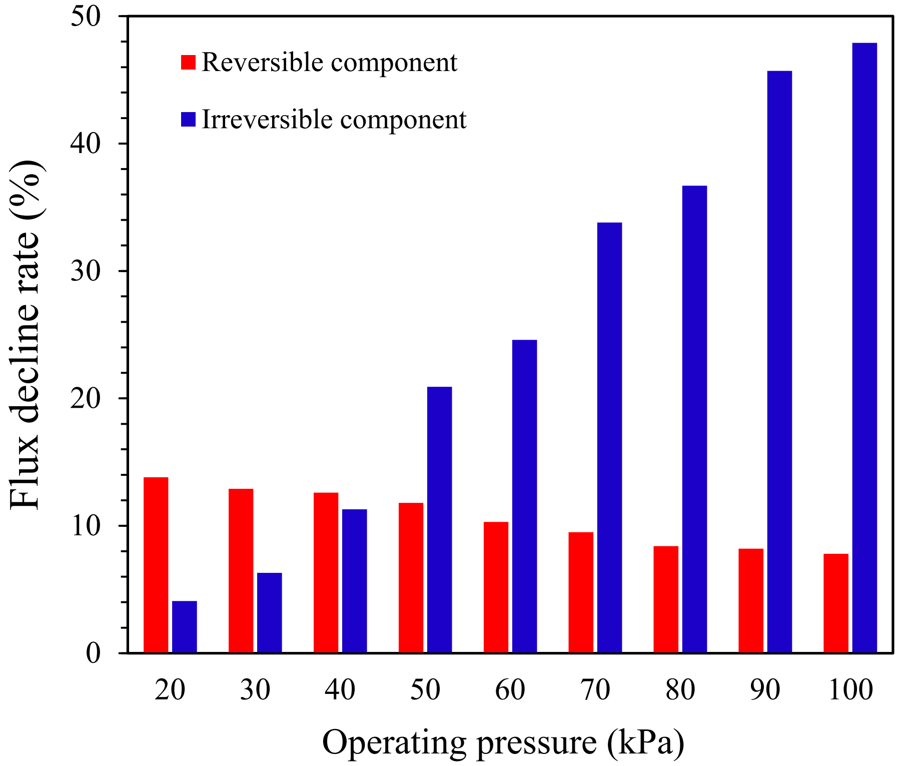

Figure S4. The reversible and irreversible flux decline rates calculated for the PES-PUMA(4.7) membrane after ten separation cycles.
